# Controlling Peptide Function by Directed Assembly Formation: Mechanistic Insights Using Multiscale Modeling on an Antimicrobial Peptide–Drug–Membrane System

**DOI:** 10.1021/acsomega.1c01114

**Published:** 2021-06-11

**Authors:** Gergely Kohut, Tünde Juhász, Mayra Quemé-Peña, Szilvia Erika Bősze, Tamás Beke-Somfai

**Affiliations:** † Institute of Materials and Environmental Chemistry, Research Centre for Natural Sciences, Magyar tudósok körútja 2, H-1117 Budapest, Hungary; ‡ Hevesy György PhD School of Chemistry, ELTE Eötvös Loránd University, Pázmány Péter sétány 1/A, H-1117 Budapest, Hungary; § ELKH Research Group of Peptide Chemistry, Eötvös Loránd University, Pázmány Péter sétány 1/A, H-1117 Budapest, Hungary

## Abstract

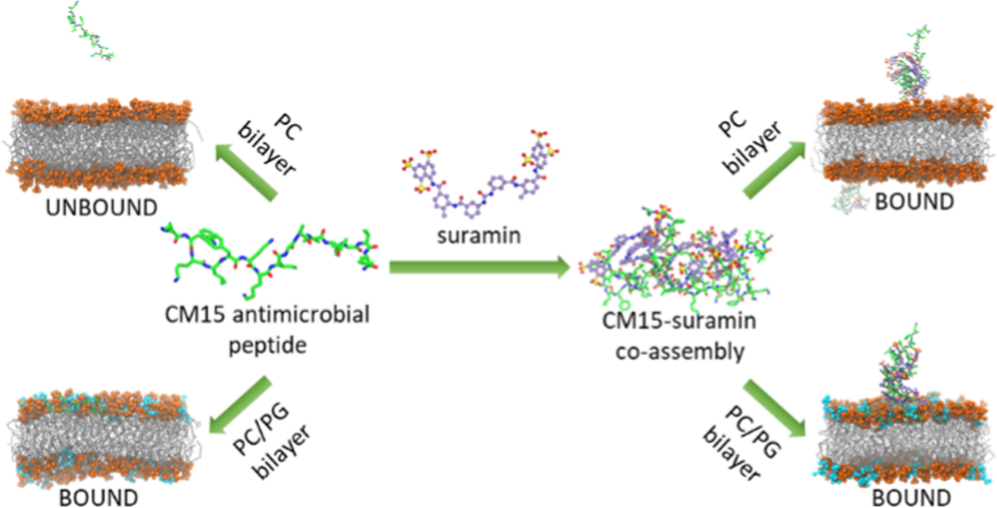

Owing to their potential applicability against multidrug-resistant bacteria, antimicrobial peptides (AMPs) or host defense peptides (HDPs) gain increased attention. Besides diverse immunomodulatory roles, their classical mechanism of action mostly involves membrane disruption of microbes. Notably, their unbalanced overexpression has also been associated with host cell cytotoxicity in various diseases. Relatedly, AMPs can be subject to aggregate formation, either via self-assembly or together with other compounds, which has demonstrated a modulation effect on their biological functions, thus highly relevant both for drug targeting projects and understanding their in vivo actions. However, the molecular aspects of the related assembly formation are not understood. Here, we focused in detail on an experimentally studied AMP–drug system, i.e., CM15–suramin, and performed all-atom and coarse-grain (CG) simulations. Results obtained for all systems were in close line with experimental observations and indicate that the CM15–suramin aggregation is an energetically favorable and dynamic process. In the presence of bilayers, the peptide–drug assembly formation was highly dependent on lipid composition, and peptide aggregates themselves were also capable of binding to the membranes. Interestingly, longer CG simulations with zwitterionic membranes indicated an intermediate state in the presence of both AMP–drug assemblies and monomeric peptides located on the membrane surface. In sharp contrast, larger AMP–drug aggregates could not be detected with a negatively charged membrane, rather the AMPs penetrated its surface in a monomeric form, in line with previous in vitro observations. Considering experimental and theoretical results, it is promoted that in biological systems, cationic AMPs may often form associates with anionic compounds in a reversible manner, resulting in lower bioactivity. This is only mildly affected by zwitterionic membranes; however, membranes with a negative charge strongly alter the energetic preference of AMP assemblies, resulting in the dissolution of the complexes into the membrane. The phenomenon observed here at a molecular level can be followed in several experimental systems studied recently, where peptides interact with food colors, drug molecules, or endogenous compounds, which strongly indicates that reversible associate formation is a general phenomenon for these complexes. These results are hoped to be exploited in novel therapeutic strategies aiming to use peptides as drug targets and control AMP bioactivity by directed assembly formation.

## Introduction

Antimicrobial peptides (AMPs or host defense peptides, HDPs, when their complex immunomodulatory roles are emphasized) are crucial compounds produced by multicellular organisms to protect the host from pathogenic microbes. They are widely considered to be a promising solution against multidrug-resistant bacteria due to their slower emergence of resistance and broad bacterial susceptibility.^1−5^ A vast majority of them have no more than 50 amino acids in their peptide sequence with a mean charge of +3 and a ∼54% average hydrophobicity.^6^ The broad-spectral activity of antimicrobial peptides is primarily associated with their structural diversity and cationic nature, but these properties can also manifest in several, sometimes controversial, modes of their mechanism of action.^7−11^ Besides the above beneficial properties, their potential cytotoxicity, sensitivity to degrading proteases, and their high production costs, at least on lower scales,^12−14^ are clear shortcomings that have to be appreciated and addressed properly. Nevertheless, despite these aspects, to date, seven of them have already been approved by the U.S. Food and Drug Administration (FDA),^6^ which clearly shows their potential as new antibiotics and makes them attractive in pharmaceutical research.

Recent studies have also indicated that AMPs can have important immunomodulatory mediators with diverse activities.^15,16^ These properties have been associated with a couple of physiological processes such as reduction of proinflammatory cytokine levels, modulation of chemokine expression, stimulation of angiogenesis, enhanced wound healing, or leukocyte activation.^17^ Furthermore, their deficiency or enhanced expression can play crucial roles in autoimmune disorders as well as in the progression of cancer, respiratory diseases, or skin diseases.^15,18−24^ Majority of their broad antimicrobial and immunomodulatory properties rely on their capability to interact with various sets of biomacromolecules. Besides their most well-known interactions with lipids of the cell membranes, there are several examples of peptide interactions with macromolecules, covering DNA,^25,26^ proteins,^27−29^ or glycosaminoglycans.^30^ Moreover, the interactions between these compounds often involve self-assembly or aggregation in a concentration-sensitive manner, however, its impact on the general mechanism and its relevance in function modulation are still far from understood.^31−34^ Recently, we have also demonstrated that various small organic compounds involving food colors, drug molecules, and other synthetic small molecules^35−40^ could also affect AMPs by controlling their structure and thereby their activity as well. Also, very similar types of interactions were observed for endogenous metabolites, phospholipid-based signaling molecules, and even for bacterial siderophores.^41,42^ All of these observations were also often accompanied by the formation of larger assemblies. Consequently, it is expected that a better understanding of associate formation could lead us a step forward toward successful pharmaceutical applications, particularly in immune regulation.

Thus, in this study, we aim to obtain a molecular-level insight into the AMP–small molecule interactions with a focus on their assemblies. As the AMP mechanism of action often involves membrane binding/disruption, we also study their interaction in a membrane environment. The hybrid AMP CM15 and the drug suramin (SUR) were chosen as a model system, as previous experimental and theoretical studies have shown that suramin was one of the potent effectors on CM15.^35,38,39,43^ CM15 (KWKLFKKIGAVLKVL) is a lysine-rich antimicrobial peptide with an overall positive (+6) charge and a synthetic hybrid of the natural AMPs cecropin A and melittin, designed to enhance the antimicrobial activity of the former and reduce the hemolytic activity of the latter.^44,45^ Suramin is a polyanionic (−6) polysulfonated naphthlyurea. It is a synthetic multipurpose drug with medical use against various diseases, including trypanosomiasis and leishmaniasis.^46−51^

Based on our previous results, we assume that the effect of suramin on peptide activity in the lipid environment is dictated by the affinities in the three-component interaction system where aggregation between the peptide and small molecule competes with membrane binding of the components. Previous studies demonstrated that due to the transient and not always well-defined structural changes in an interacting membrane–peptide–small-molecule system, unfortunately, experimental structure-determining methods cannot provide sufficient insight. However, computational simulations have the advantage of reporting on these processes. Thus, in this study, we investigate our model system primarily by all-atom and coarse-grained (CG) molecular dynamics (MD) methods, emphasizing CM15–suramin aggregation.

Accordingly, the interactions between CM15 and suramin were studied by MD in three environments: aqueous phase, aqueous phase in the presence of phosphatidylcholine (PC) bilayer representing the outer leaflet of a mammalian cell, and aqueous phase in the presence of phosphatidylcholine–phosphatidylglycerol (PC/PG) bilayer mimicking charge properties of a bacterial inner cell membrane. The two-component peptide–drug system in the aqueous phase reflects on the general capability of suramin–CM15 aggregation, while the simulations in PC and PC/PG bilayers address the impact of suramin on the membrane activity of CM15 on model lipid bilayers. Note that these are rather simplistic models of complex natural environments. Nevertheless, they can still provide valuable insight into the underlying mechanism and the conceptual differences observed for the corresponding experimental findings.

Furthermore, since some calculations here focused on suramin–membrane interactions, for which we are not aware of experimental details, we have also performed experiments addressing interactions and relative orientation of suramin on PC and PC/PG bilayers. This is of particular interest in the currently selected example, as the 100-year-old drug suramin was subject to several successful repurposing strategies, where some targets are intracellular, beyond cell walls.^52^ Moreover, it even shows promise against the current SARS-CoV-2 infection by inhibiting key interactions during viral reproduction,^53,54^ however, its affinity to lipid bilayers is not explored. To this end, linear dichroism (LD) measurements were used, a technique suitable to study systems that are either intrinsically oriented or can be oriented by external forces. LD can give information about membrane insertion, orientation angle, and structure of associated molecules.^55−60^ Additionally, supportive quantum mechanical (QM) calculations were performed to correlate the orientational information of LD measurements with the corresponding transition dipole moments (TDMs) of suramin.

## Methods

### All-Atom Simulations

#### All-Atom Simulation Protocols

**Figure 1:**
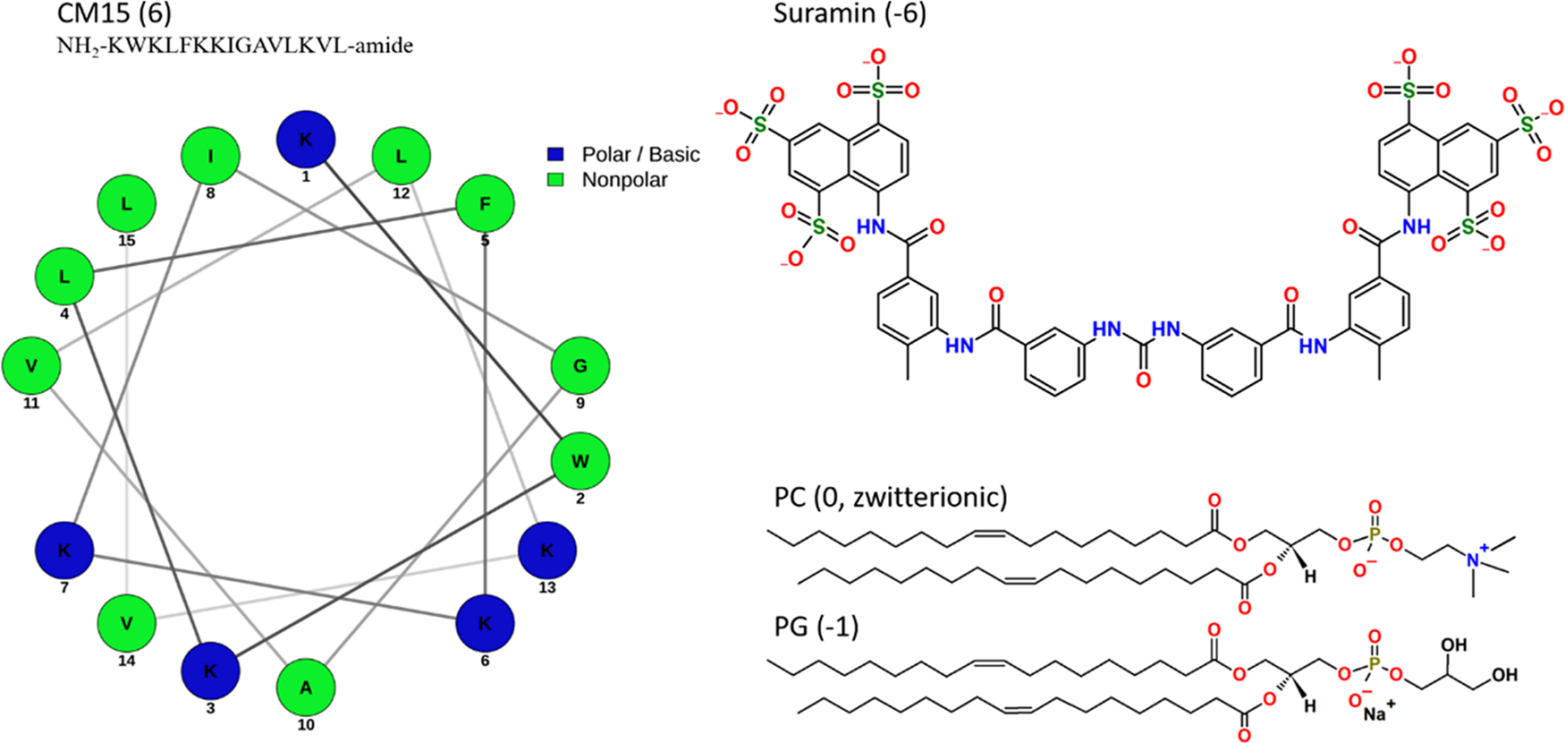
Structures of the main molecules studied here. The numbers in parenthesis indicate the charge of the compounds under physiological conditions. For peptide CM15, the helical wheel model is also displayed, demonstrating the relative position of the amino acids in the peptide sequence.

The CM15–suramin system was first studied at an atomistic level in three environments: in the aqueous phase and near PC and PC/PG bilayers mimicking mammalian and bacterial cell membranes, respectively (Figure 1 and Table 1).

**Table 1:** Number of CM15 and Suramin Molecules in the Different Environments

	CM15/suramin			
	1	2	3	4
water			1:1	4:4
PC	0:1	1:0	1:1	4:4
PC/PG	0:1	1:0	1:1	4:4

Here, we addressed determinants of the CM15–suramin aggregation and the impact of different lipid bilayers on it; thus, the water phase behavior of the sole molecules was not investigated. Also, we focused on equimolar CM15–suramin systems, as considering other molar ratios would significantly increase the scenarios to be studied.

The simulations were carried out using the GROMACS 2018.3^61^ package and the CHARMM36m^62^ force field. Parameters for suramin were generated by CgenFF v4.4.0^63,64^ and converted from CHARMM to GROMACS format using the charmm2gmx python script provided by the MacKerell Lab.

The initial disordered CM15 and suramin structures were taken from simulations of our previous study.^38^ The molecules were initially placed 3 nm away from each other, while in the case of the 4:4 CM15–suramin simulation, the molecules were placed on the edges of a rectangular box. For aqueous phase simulations, the boxes were then solvated with TIP3P water molecules, and 150 mM NaCl was added to mimic the physiological ion concentration. For the bilayer-containing simulations, first, solvated bilayers with 128 lipids in each leaflet were generated using CHARMM-GUI.^65^ The pure PC bilayer consisted of 1,2-dioleoyl-*sn*-glycero-3-phosphocholine (DOPC) lipids, and the PC/PG bilayer contained 80% DOPC lipids and 20% 1,2-dioleoyl-*sn*-glycero-3-[phosphorac-(1-glycerol)] (DOPG) lipids. Then, solvated boxes containing various CM15–suramin compositions were placed on top of the bilayer with the same *a* and *b* cell unit parameters as the bilayer box. The merged systems were neutralized, and Na^+^ and Cl^–^ ions were added at 150 mM concentration.

Both the aqueous phase and bilayer-containing systems were minimized and equilibrated in six steps. The energy minimization was done using the steepest descent algorithm in 5000 steps with a maximum force tolerance of 1000 kJ mol^–1^ nm^–1^. In the following two steps, the systems were heated up using Berendsen and V-rescale thermostats^66,67^ for 75 ps (300 K temperature, τ_T_ = 1.0 ps). In the remaining steps, the systems were equilibrated for 650 ps as an NPT ensemble coupled to a V-rescale thermostat in 300 K temperature (τ_T_ = 1.0 ps), and a semi-isotropic Berendsen barostat^66^ (except for the aqueous phase simulations, where isotropic coupling was applied) of 1 bar pressure (τ_P_ = 5.0 ps, κ_P_ = 4.5 × 10^–5^ bar^–1^). The atomic position restraints were decreased and switched off gradually during the six steps (Table S1).

To ensure that the correct statistical ensemble was sampled, 500 ns production runs were carried out using a Nosé–Hoover thermostat^68^ with 1.0 ps coupling time and a semi-isotropic/isotropic Parrinello–Rahman barostat^69^ with 5.0 ps coupling time. For all production runs, a 2.0 fs time step size was used, the electrostatics were treated by the particle-mesh Ewald method,^70^ the cutoff was 1.2 nm, and a Verlet cutoff scheme was applied. The LINCS algorithm^71^ was used to constrain the hydrogen bonds. Periodic boundary conditions were applied in all directions.

### Coarse-Grained Simulations

#### Simulation Protocols

The suramin molecule was parametrized using the β version of the MARTINI v3.0^72^ force field. It was used instead of the latest MARTINI v2.2^73^ due to its better performance during parameter validation when applied to the mapping (Figure 2). Extended details on parameterization and validation process are given in Section I.2 (Figures S1–S16) of the Supporting Information (SI).

**Figure 2:**
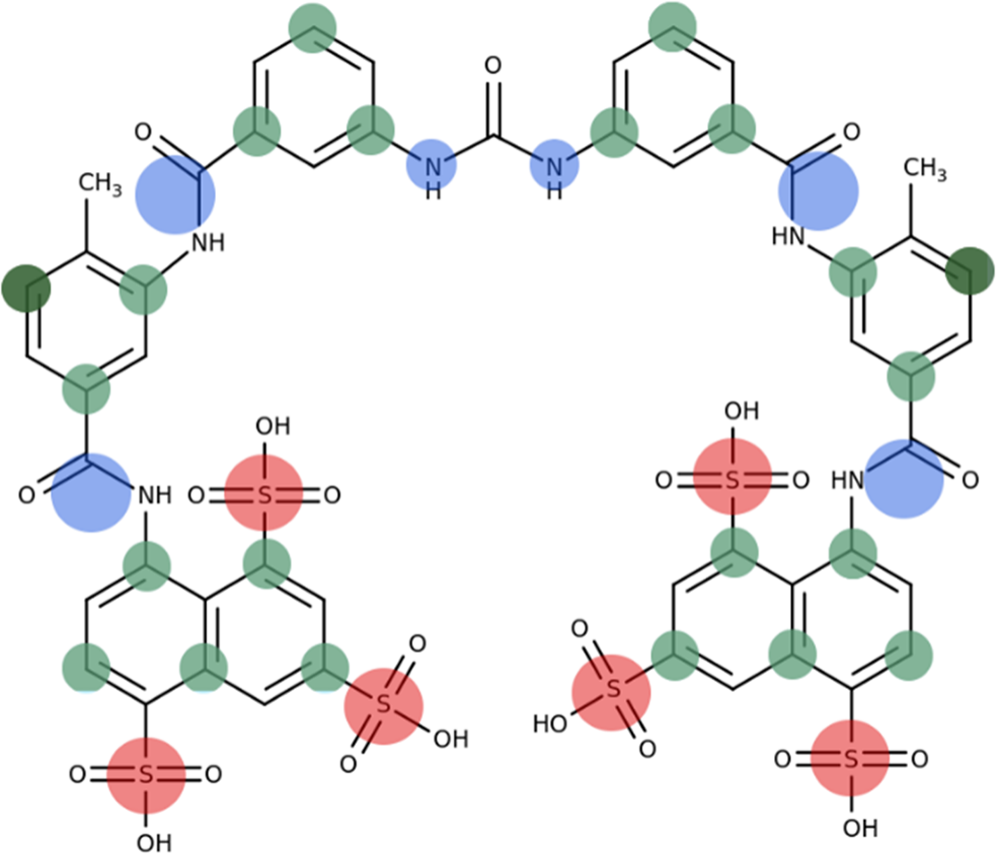
Atom-to-bead mapping of suramin based on the MARTINI v3.0 force field. Different colors show different MARTINI v3.0 bead types: red, SQ1; light green, TC5; dark green, TC4; blue, SP4.

Coarse-grained simulations were performed with GROMACS 2018.3^74^ and the MARTINI v3.0^72^ force field to be consistent with the suramin parametrization. CM15 and suramin initial structures used in the all-atom simulations were coarse-grained via the martinize Python script provided by MARTINI developers. The initial structures were then merged and replicated using the GROMACS genconf command to create a system with 36 CM15 and 36 suramin molecules. The system was then placed at the center of a rectangular box. The box was solvated with water molecules, and Na^+^ and Cl^–^ ions were added at 150 mM concentration. The system was minimized using the steepest descent algorithm for 50 000 steps and was heated up to 300 K temperature using a V-rescale thermostat (τ_t_ = 0.5 ps) in 10 ns. It was then followed by an NPT simulation of 50 ns, where the system was additionally coupled to an isotropic Berendsen barostat with 1 bar pressure (τ_p_ = 6.0 ps, κ_P_ = 4.5 × 10^–4^ bar^–1^). Production runs were carried out using a V-rescale thermostat (τ_t_ = 1.0 ps) and an isotropic Parrinello–Rahman barostat (τ_p_ = 16.0 ps, κ_P_ = 3.0 × 10^–4^ bar^–1^) for 10 μs simulation time. Coulomb interactions were treated by the reaction-field method, and a Verlet cutoff scheme was used. The simulation time step was 20 fs for all simulations.

For the bilayer-containing simulations, the bilayers were created using the insane Python script provided by MARTINI developers. Both the PC and PC/PG bilayers contained 3195 × 3195 lipids in the two leaflets. The PC bilayer was built up by DOPC lipids, and the lipid ratio of the PC/PG bilayer was 80% DOPC and 20% DOPG lipids, consistent with the all-atom simulations. The generated bilayers were first minimized and equilibrated for 1 μs, and the last structure of the trajectory was used as a starting point for the CM15–suramin–bilayer simulations. The 36 suramin and 36 CM15 molecules were replicated and merged in the same way as described above. The merged systems were solvated and placed above the bilayers. The merged, suramin-, CM15- and bilayer-containing systems were neutralized, and the ion concentration was set to 150 mM by the addition of Na^+^ and Cl^–^ ions. The systems were then subjected to the same minimization, equilibration, and production processes as described above, except the usage of isotropic pressure coupling, which was modified to be semi-isotropic.

#### Free Energy Calculations

The free energy differences along the distance-based collective variables (potential of mean force, PMF) were calculated using umbrella sampling^75^ and Plumed 2.4.3^76^ software. The distances were defined through the center of mass (COM) of the compounds. The bilayer surface was defined by the COM of the phosphorous atoms of the containing lipids. The distance from the bilayer surface was defined by the *z* coordinate difference between the COM of the bilayer and the COM of CM15/suramin. A distance of 5 nm was covered by a sum of 50 windows, which represented a 0.1 nm step size. A standard quadratic potential was used with κ = 1000 kJ mol^–1^ nm^–2^. In the case of the all-atom simulations, each window was simulated for 25 ns, and the coordinates were saved in 0.1 ps, and the last 20 ns were used for PMF calculation (see Figures S17 and S18 for window distributions). Regarding the coarse-grained simulations, each window was simulated for 100 ns, and the coordinates were saved with 1.0 ps step size, and the last 75 ns were used for PMF calculations. Other parameters were the same as the ones used in the corresponding MD level. The PMF was calculated at 300 K temperature applying the vFEP approach.^77^

#### Calculation of the Aggregation Evolution

The time dependence of aggregate growing was calculated in the following way. A trajectory was first generated with 1000 frames, defining 10 ns steps in the simulation. The CM15 and suramin molecules were then clustered using the DBSCAN^78^ approach in each frame. The distance metric was the Euclidean distance between the center of mass of the molecules, ε was set to 15.0 Å, the minimum points were chosen to be 2. Each cluster was then considered a separate aggregate, and the molecules of the biggest aggregate in the last frame were identified. Then, the molecular overlaps between the above-identified aggregate and the aggregates of the previous frame were determined, and the aggregate with the biggest overlap was considered the predecessor of the current aggregate. The composition difference between the current and the predecessor aggregate was defined as the growth or shrink of the selected aggregate. The iterative repetition of the above-described backtracking process was used to follow the time dependence of the aggregate formation.

### Quantum Chemical Calculations

Gaussian 16 software^79^ was used for all of our calculations. The initial structure of the suramin was taken from our all-atom MD simulations. Due to our assumption that the central part of the suramin is responsible for bilayer binding, the sulfonated naphthyl rings were substituted with hydrogen atoms to make it more feasible for QM calculations. The structure was optimized in vacuum by the density functional theory (DFT) method using the B3LYP functional and applying the 6-31+G(d) basis set. The excited states were calculated on the optimized structure by the time-dependent DFT (TD-DFT) method applying the 6-311++G(d,p) basis set and using the CAM-B3LYP functional, which has been proven to outperform the standard B3LYP functional in excited state calculations.^80^ To better represent the molecular orbitals (MOs) involved in the excitation, the natural transition orbitals (NTOs) of selected excited states were calculated from the canonical MOs.

### Experimental Methods

#### Suramin and Lipid Solutions

Suramin sodium salt (≥99%, S2671) was purchased from Sigma-Aldrich (Hungary), dissolved in high-purity water at 1 mM, aliquoted, and stored at −18 °C until use. High-purity synthetic 1,2-dioleoyl-*sn*-glycero-3-phosphocholine (DOPC) and 1,2-dioleoyl-*sn*-glycero-3-[phosphorac-(1-glycerol)] (DOPG) were purchased from Avanti Polar Lipids, Inc. Unilamellar vesicles (100 nm) of pure PC and 80:20% n/n DOPC/DOPG (80:20%, n/n) were prepared as described in our previous studies.^39^

#### Linear Dichroism (LD) Spectroscopy

Linear dichroism measurements were performed on a JASCO-1500 spectropolarimeter equipped with a Couette flow cell system (CFC-573 Couette cell holder). LD and absorbance spectra were recorded in phosphate-buffered saline (PBS) supplemented with 50 wt % sucrose between 195 and 400 nm at a rate of 100 nm min^–1^ with a data pitch of 0.5 nm, a response time of 1 s, a bandwidth of 1 nm, and a total path length of 0.5 mm. Suramin and lipid concentrations were 260 μM and 1.2 mM, respectively. For LD, samples were oriented under a shear gradient of 2270 s^–1^, and spectra measured at zero shear gradient were subtracted. Absorbance spectra were calculated by direct conversion of the recorded HT data. For the suramin solution (10 μM), absorbance spectra were also recorded on a Hewlett-Packard 8453 diode array spectrophotometer using a quartz cuvette with a 1 mm optical path and were normalized to parameters used in LD experiments.

## Results and Discussion

### All-Atom Simulations

A prerequisite for investigating the three-component systems is to gain a better insight into the behavior of the two-component CM15–suramin, CM15–PC, CM15–PC/PG, suramin–PC, and suramin–PC/PG systems. The aqueous phase interactions between CM15 and suramin and the potential impact of suramin on the structure of CM15 were recently studied in detail in our previous paper.^38^ In that study, we have provided a detailed analysis and insight at the molecular level on how suramin impacts the secondary structure of CM15 by increasing its helicity, in line with experimental observations. However, in related studies,^39^ it has also been demonstrated that suramin changes membrane behavior of this AMP, which is a crucial phenomenon in understanding the effect of co-assembly formation on antimicrobial activity and AMP–membrane interactions. Consequently, as a further step in understanding this complex process, we aimed to study the association ability of the CM15–suramin system and how the surrounding environment may influence this co-assembly formation. Thus, in the next section, we aim to focus on the following four systems identified to be essential for such an investigation.

**Figure 3:**
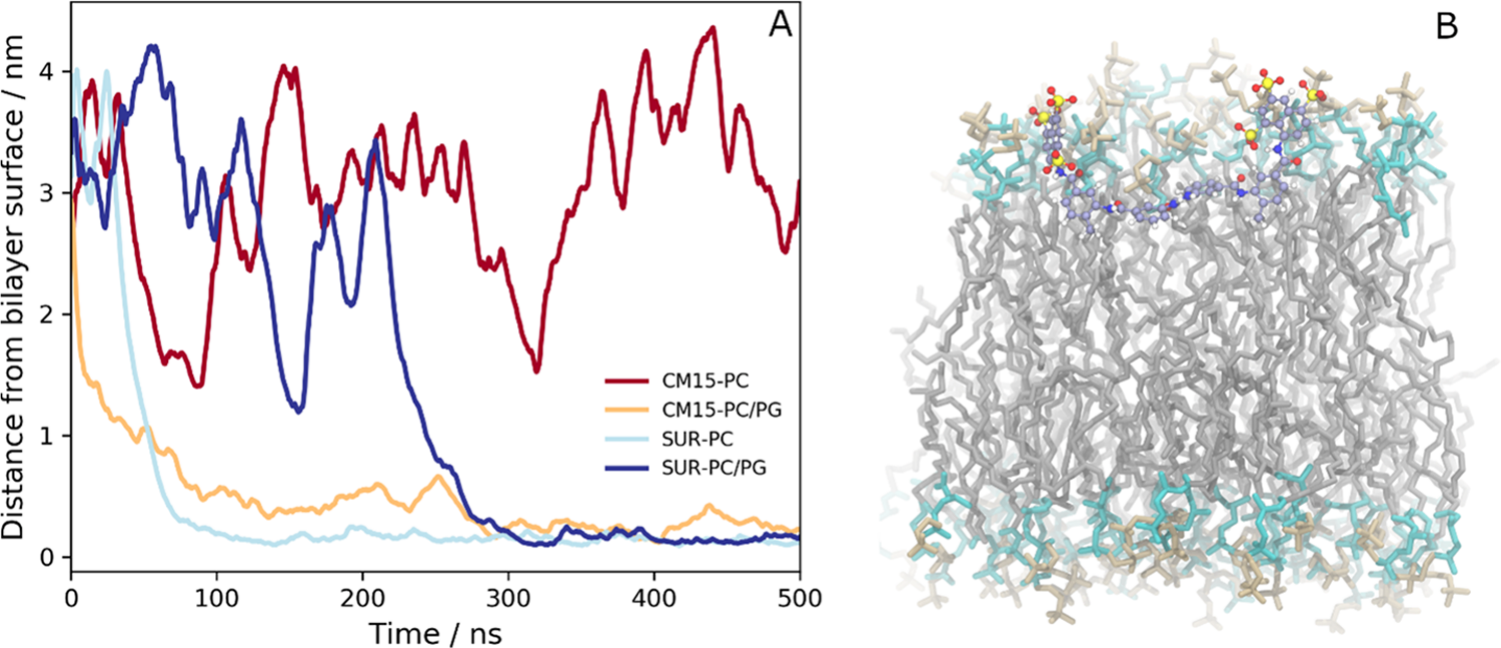
Simulation results of the two-component systems. (A) Surface distances of CM15 and suramin in different environments. Surface distance is defined as the modulus of the minimum value of the *z* coordinate differences between the corresponding top and bottom surfaces of the bilayer and the center of mass (COM) of the particular CM15 or suramin molecule. The COM of the surfaces was calculated based on the phosphorous atoms of the lipids in the top and bottom leaflets, respectively. For more details, please see the text and also section “Pairwise Comparison of the Components through Free Energy Calculations and Experimental Findings.” (B) Snapshot (400 ns) of the suramin bound to the PC bilayer. Lipid alkyl chains are shown in gray, glycerol parts in cyan, and choline head groups in light brown. Suramin carbon atoms are shown in purple, nitrogen atoms in blue, sulfur atoms in yellow, and oxygen atoms in red.

These systems showed remarkable differences in their interaction profiles, which were primarily assessed by the distance between CM15 or suramin molecules and the bilayer surfaces (Figure 3).

We have found that peptide CM15 almost immediately binds to and, after a while, partially penetrates the head group region of the PC/PG membrane. In contrast, it remains in the aqueous phase in the presence of the PC bilayer for the whole course of the simulation. This is an expected result if we trace it back to the electrostatic interactions of oppositely charged molecules, which is commonly considered to be highly important in the mechanism of action of AMPs and is also in line with previous MD results.^81^

However, the results of suramin simulations are far less expected if we assume the dominance of the electrostatic interactions, as suramin has bound to both the PC and PC/PG bilayers, even though it carries a net negative charge of −6. On the one hand, the reasons for this rather unexpected behavior lies in the amphipathic nature of suramin due to the hydrophobic central core vs the hydrophilic terminal parts. Consequently, the hydrophilic–hydrophobic boundary of the outer leaflet of a lipid bilayer can provide an ideal binding site for suramin, facilitating its binding. However, this probably would not be enough, as suramin is highly negatively charged, its binding would increase the negative charge density of the surface layer, resulting in unfavored electrostatic repulsion and thereby decreased stability. Note that the positively charged PC choline head groups could shield the electrostatic repulsion between the sulfonated naphthyl rings of the suramin by the formation of cation−π interactions with them, allowing suramin binding even to a bilayer containing negatively charged PG lipids (Figure 3B). The involvement of choline groups is supported by the short choline–naphthyl distances, being 0.60 nm (STD 0.79 nm), 0.69 nm (STD 0.80 nm), 0.94 nm (STD 0.76 nm) for the three closest cholines, respectively (Figure S19).

**Figure 4:**
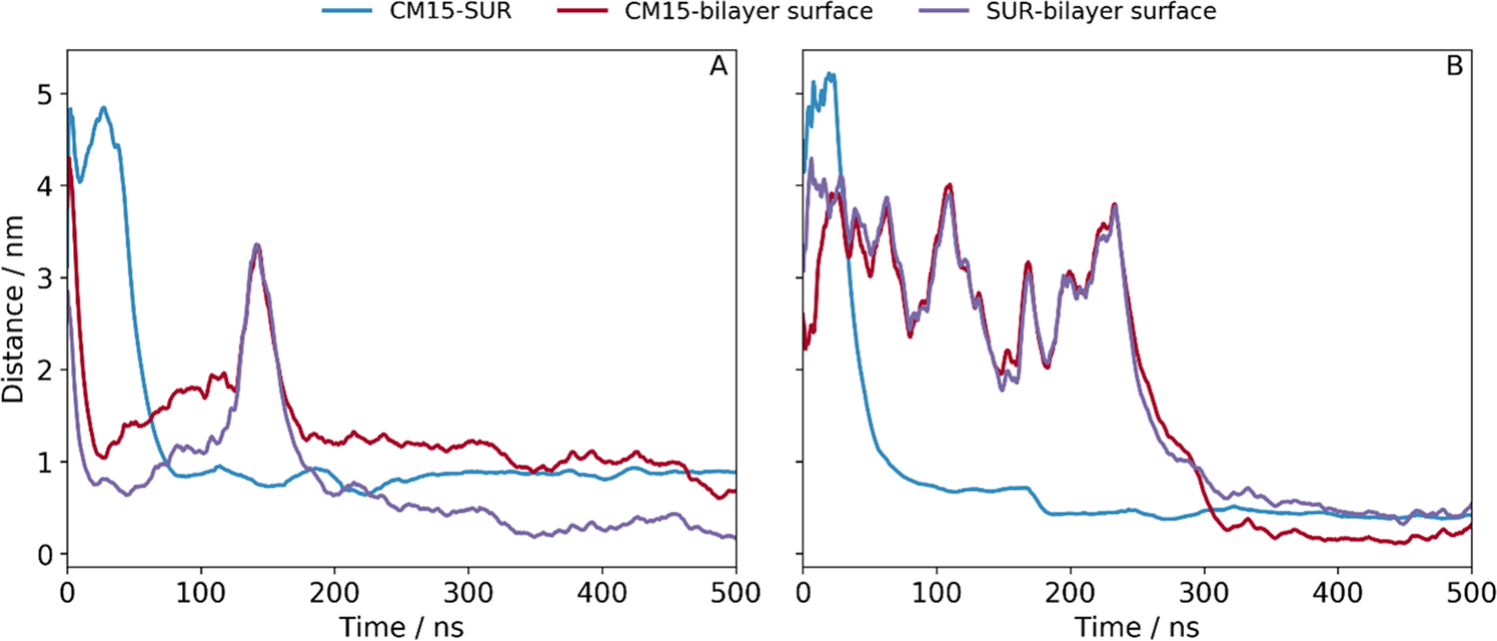
Bilayer distances as a function of the simulation time. (A) Distance between the CM15, suramin, and PC bilayer surface. (B) Distance between CM15, suramin, and PC/PG bilayer surface.

Based on the analysis of the simulations on the two-component systems and our previous results, both the peptide CM15 and the drug suramin can interact with lipid bilayers and with each other; hence, in the next step, we carried out simulations on these rather competitive three-component systems to reveal binding preferences. As done for the two-component systems above, pairwise distances were first calculated and analyzed (Figure 4).

In the PC–peptide–drug system, right after the beginning of the simulation, both CM15 and suramin molecules get close to the PC bilayer, which is indicated by a rapid decrease in their bilayer surface distance (Figure 4A). After ∼30–40 ns, CM15 and suramin form a complex as the rapid drop in the distance between them shows. The stable complex stays near the PC bilayer surface in the majority of the following 100 ns. After that, the complex partially inserts into the bilayer surface with its suramin side facing the membrane and remains there for the whole course of the simulation, as suggested by the decreased suramin–bilayer distance and the constant CM15–suramin distance.

Similar to the PC system, the CM15 and suramin molecules bind together at the beginning of the simulation in a PC/PG environment too, but the complex remains in the bulk phase for the next 200 ns (Figure 4B). However, in the second half of the simulation, the complex binds to the bilayer surface with its “CM15 face” toward the membrane, which remains unvaried for the rest of the simulation. To sum up, it was revealed that the individual molecules and the complex could bind and partially insert into the bilayer in both lipid environments.

As the next step of the all-atom simulations, we performed simulations with 4:4 CM15–suramin molecules in an aqueous phase, PC, and PC/PG bilayer environments. The aqueous phase simulation showed a rapid aggregation process, resulting in one big aggregate incorporating all peptide and drug molecules (Figures S20 and S21). This indicated that the aggregation is highly favored in the solvent phase, even though the rate might be faster than in a corresponding experimental setup due to the high concentration of the molecules, which are necessary to perform the particular simulation.

**Figure 5:**
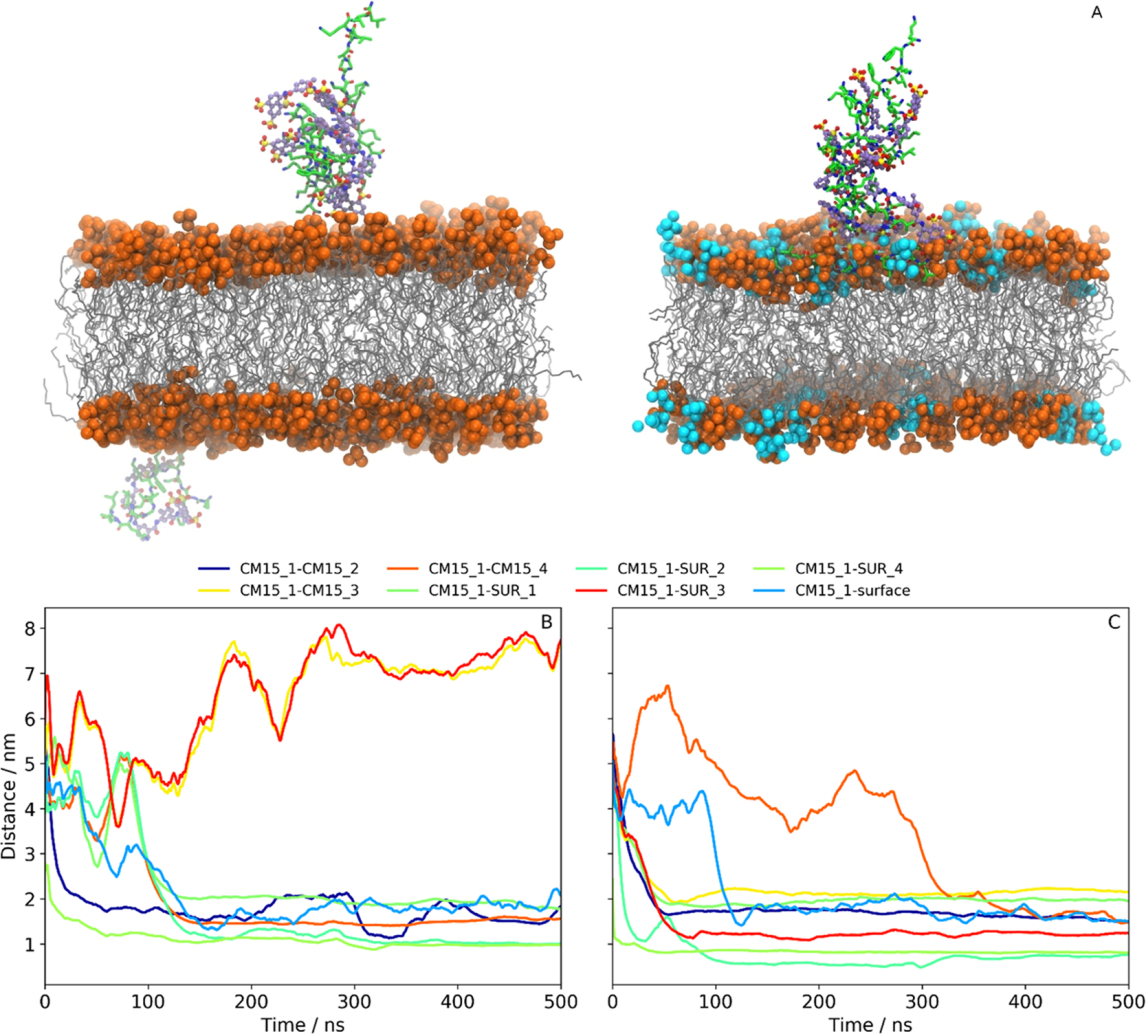
Simulation results of the 4:4 CM15–suramin systems in lipid environments. (A) Snapshots of the molecules in the PC (left) and PC/PG (right) environment. Lipid head groups are represented by orange beads, and lipid alkyl chains are shown in gray. CM15 carbon atoms are shown in green, nitrogen atoms in blue, and oxygen atoms in red. Suramin carbon atoms are shown in purple, nitrogen atoms in blue, sulfur atoms in yellow, and oxygen atoms in red. (B) Distances between one selected CM15 and all other molecules, including the distance from the PC bilayer surface. (C) Distances between one selected CM15 and all other molecules, including the distance from the PC/PG bilayer surface.

In the lipid environments, the 4:4 CM15–suramin simulations displayed characteristics like those observed for the 1:1 system. The binding processes detected involved again the formation of peptide–drug aggregates and their binding to both PC and PC/PG bilayers, however, with notable differences (Figure 5A). In the presence of pure PC, two aggregates were formed in the first 100 ns (1:1 and 3:3 CM15/suramin), which remained bound to the opposite surface layers (Figures 5B, S22, and S23). On the contrary, in the case of the PC/PG membrane, first, a single CM15 molecule bound and became embedded into the bilayer, followed by the formation of a 3:4 CM15–suramin aggregate (Figures 5A, 4C, S24, and S25). This aggregate eventually attached to the surface through binding to the already inserted CM15 molecule, which resulted in a 4:4 CM15–suramin associate. Note that there is a qualitative agreement between the 1:1 and 4:4 simulations in that for PC first again the “suramin face” of the assemblies approached the membrane, whereas for the PC/PG bilayer, the first contact to the surface was made by the AMP.

### Coarse-Grained Simulations

**Figure 6:**
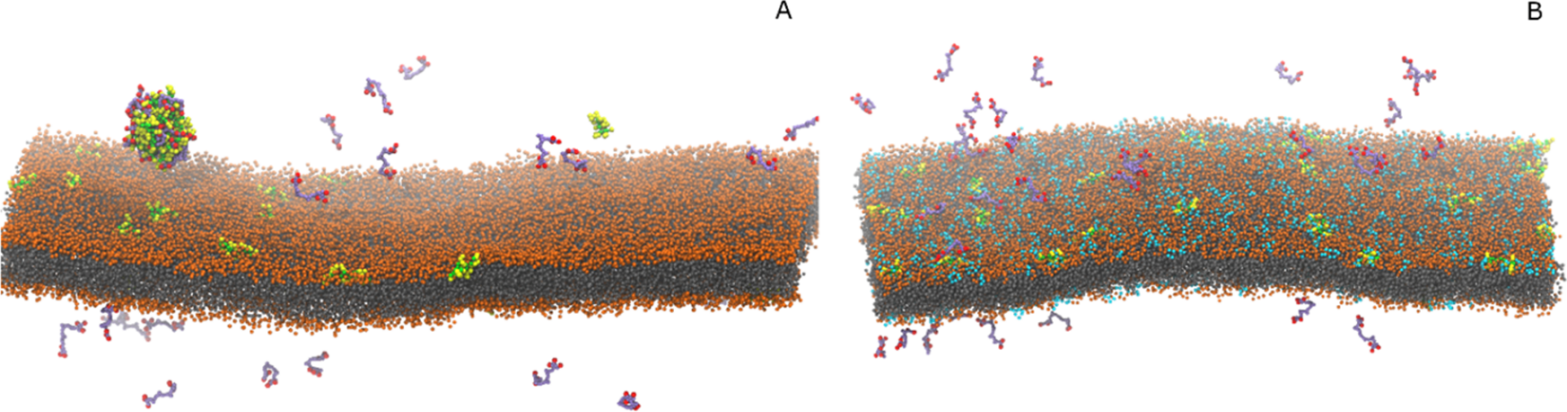
Snapshots of the molecules (A) in PC and (B) PC/PG environment at the end of the CG simulations. Suramin molecules are represented by purple and red beads, CM15 backbone beads are in green, residues are in yellow, PC and PG head groups are orange and cyan beads, respectively, and the acyl chains are in gray.

All-atom simulations provided valuable insights into the characteristic interactions of the studied systems; nevertheless, it is not feasible to simulate molecular systems with a high number of molecules on a microsecond timescale, which is essential to study the dynamic processes of aggregation. Therefore, we turned to the coarse-grained level and carried out 10 μs many-molecule computations, and to assess suramin impact on CM15 membrane activity, simulations were performed in the same environments as in the all-atom simulations: in the aqueous phase and the presence of PC or PC/PG lipid bilayers (Figure 6).

**Figure 7:**
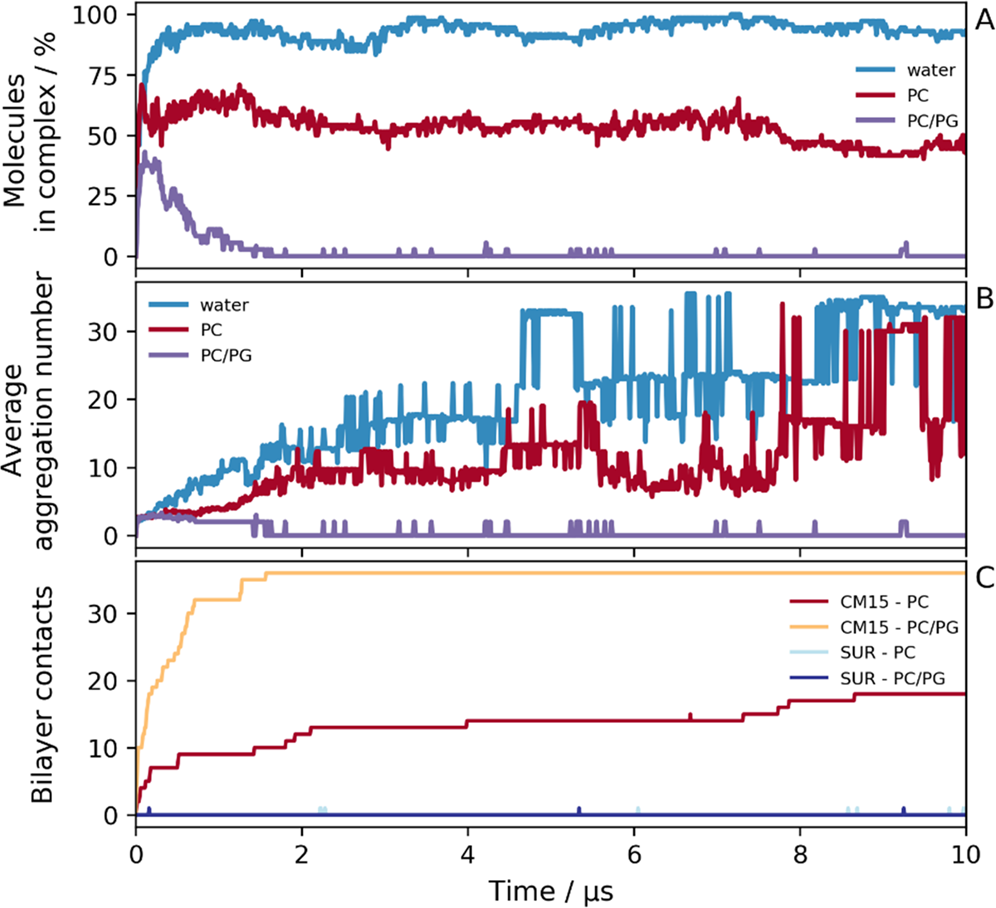
Time dependence of CM15–suramin aggregation in three different environments. (A) Ratio of the number of molecules in complex relative to the total number of molecules (36 CM15 and 36 suramin), (B) average aggregation number of the aggregates, which is the fraction of the total number of molecules and the number of aggregates, and (C) total number of suramin and CM15 molecules in contact with the various lipid bilayers is shown.

Similar to the all-atom MD simulations, the aggregation was significantly affected by the surrounding environment (Figure 7). In the aqueous phase, CM15–suramin aggregates formed almost immediately and completely, and, although with some fluctuation, the molecules remain complex for the duration of the 10 μs simulation (Figure 7A). As indicated by the aggregation number, the initially smaller multimolecular complexes assembled to bigger ones, which concluded in large aggregates with an average aggregation number of 34–35 molecules by the end of the simulation (Figure 7B). On the other hand, in the presence of the negatively charged PC/PG lipid bilayer, besides some complex formation at the beginning of the simulation, there is no indication of suramin–CM15 aggregation, as illustrated by the time dependence of the ratio of the molecules in complex and the average aggregation number (Figure 7A,B). Here, the CM15 molecules rapidly bind to the bilayer, and the system reaches a saturated state after ∼1.8 μs, where all of the 36 CM15 molecules are bound to the PC/PG bilayer (Figure 7C). The strong interaction of the CM15 molecules with the lipids prevents them from forming aggregates with suramin molecules; hence, the latter remains free for the subsequent time of the simulation.

**Figure 8:**
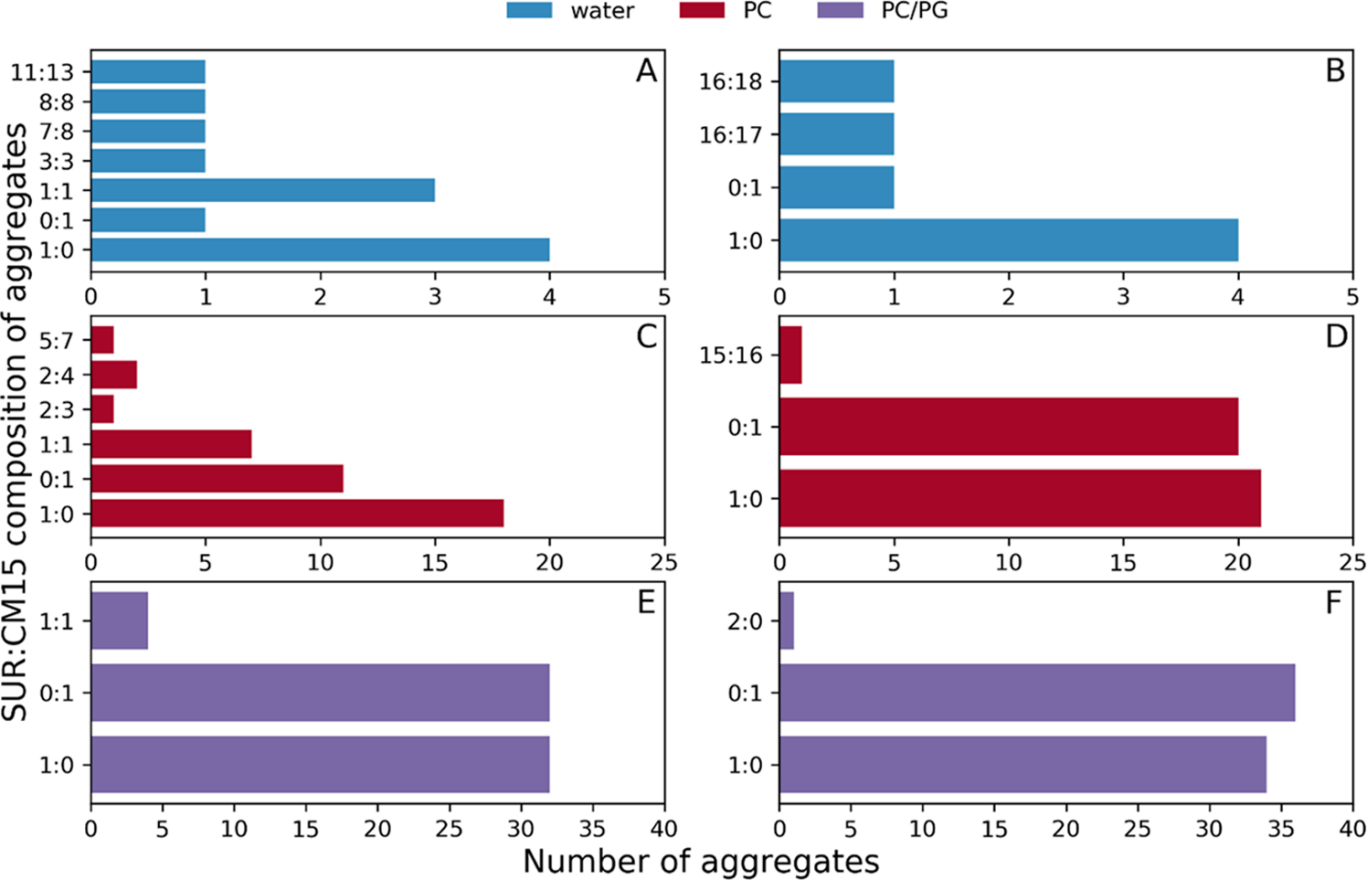
SUR/CM15 composition of the formed aggregates. (A, C, E) after 1 μs and (B, D, F) after 10 μs in three different environments. The number of free molecules (0:1 for CM15 and 1:0 for SUR) is also shown for better clarity.

Most interestingly, the simulation with the PC bilayer represents an intermediate situation between the above two setups. Although some CM15 molecules bind to the bilayer, others prefer to form aggregates, as demonstrated by the ratio of molecules in complex to the total number of molecules and the number of bilayer contacts (Figure 7A,B). This also suggests the competitive nature of aggregation and bilayer binding. It is worth noting that the average aggregation number of the solvent-phase CM15–suramin aggregates increases over time, which could mean that the aggregation takes effect somewhat similar to the pure water simulation (Figure 7B). This similarity becomes even more apparent when the composition of the formed aggregates is analyzed in detail (Figure 8).

When comparing the three different CG setups with each other, for the simulations in aqueous solution and with a PC bilayer, the aggregates formed similarly, though to a lesser extent for the latter. After 1 μs simulation time, the formation of medium-sized aggregates can be identified with multiple smaller ones in both cases, which then concludes in one to two large aggregates with a similar composition (Figure 8A,D). Note that charge neutralization seems to dominate the interactions as the formed aggregates tend to have a close to an equal number of suramin and CM15, even though a small excess of CM15 molecules over suramin can be identified in the aggregates. The fact that the final aggregates have an aggregation number of 33–34 in both simulations suggests that this aggregate size is ideal, and further aggregation is not preferred, or it takes place on a different timescale. At the same time, some CM15 molecules remain bound to the membrane even by the end of the simulation. In contrast, in the case of the PC/PG setup, only one single complex is formed, which then decomposes to monomers (Figure 8E). All of these are in line with results obtained from the analysis of the complex ratio, average aggregation number, or bilayer contacts.

**Figure 9:**
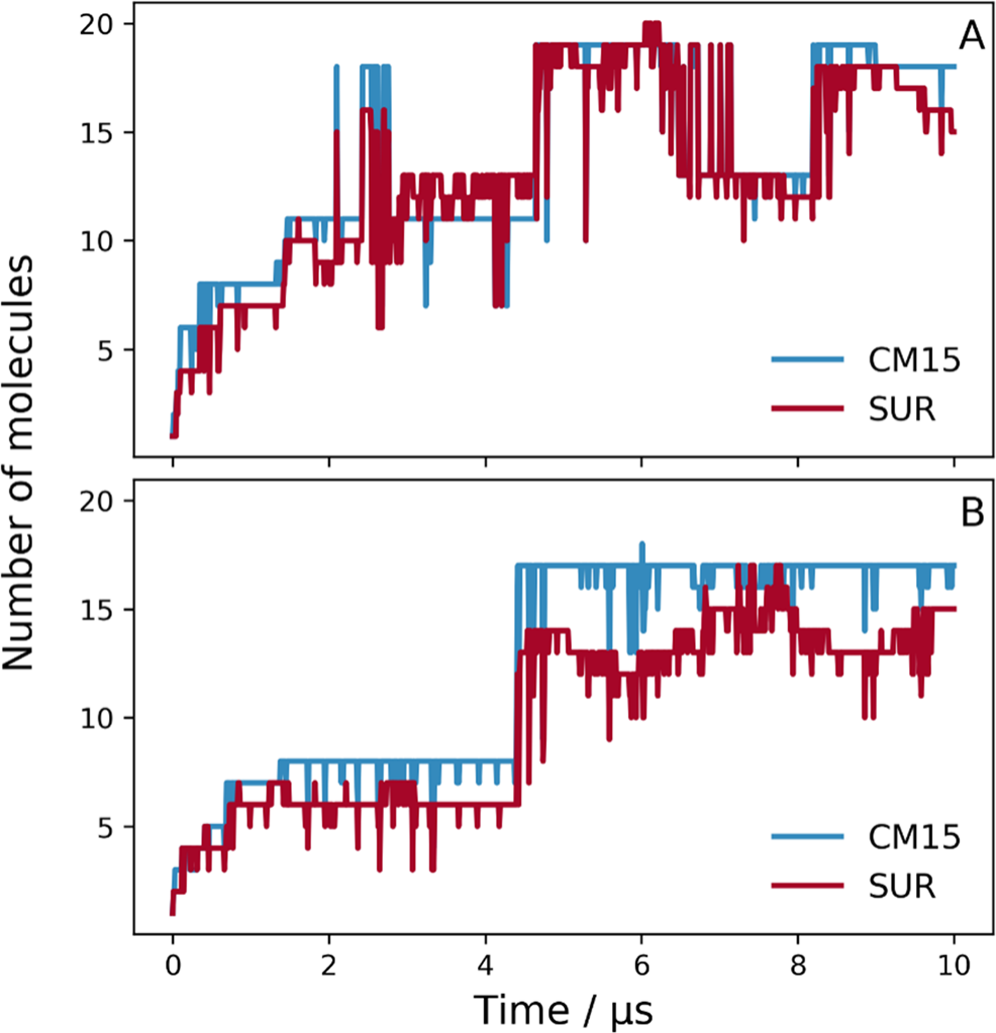
Time dependence of aggregate growth (A) in water and (B) in the PC environment.

To further characterize the nature of aggregate formation, the time dependence of aggregate growth was calculated for one selected aggregate, accumulating finally a high number of peptide and suramin molecules (see the Calculation of the Aggregation Evolution section in Methods) in water and with PC bilayer (Figure 9), which lead to three major observations. First, the huge steps in the curve indicate that aggregation is driven via collision-based fusion of smaller associations instead of continuous growth by subsequent binding of free suramin and CM15 monomers. Second, while the association is the leading process, dissociation also occurs, which is a clear indication of reversibility. Third, an excess of CM15 over suramin in the aggregate composition was observed for the entire simulation time, especially in the case of PC.

### Pairwise Comparison of the Components through Free Energy Calculations and Experimental Findings

**Figure 10:**
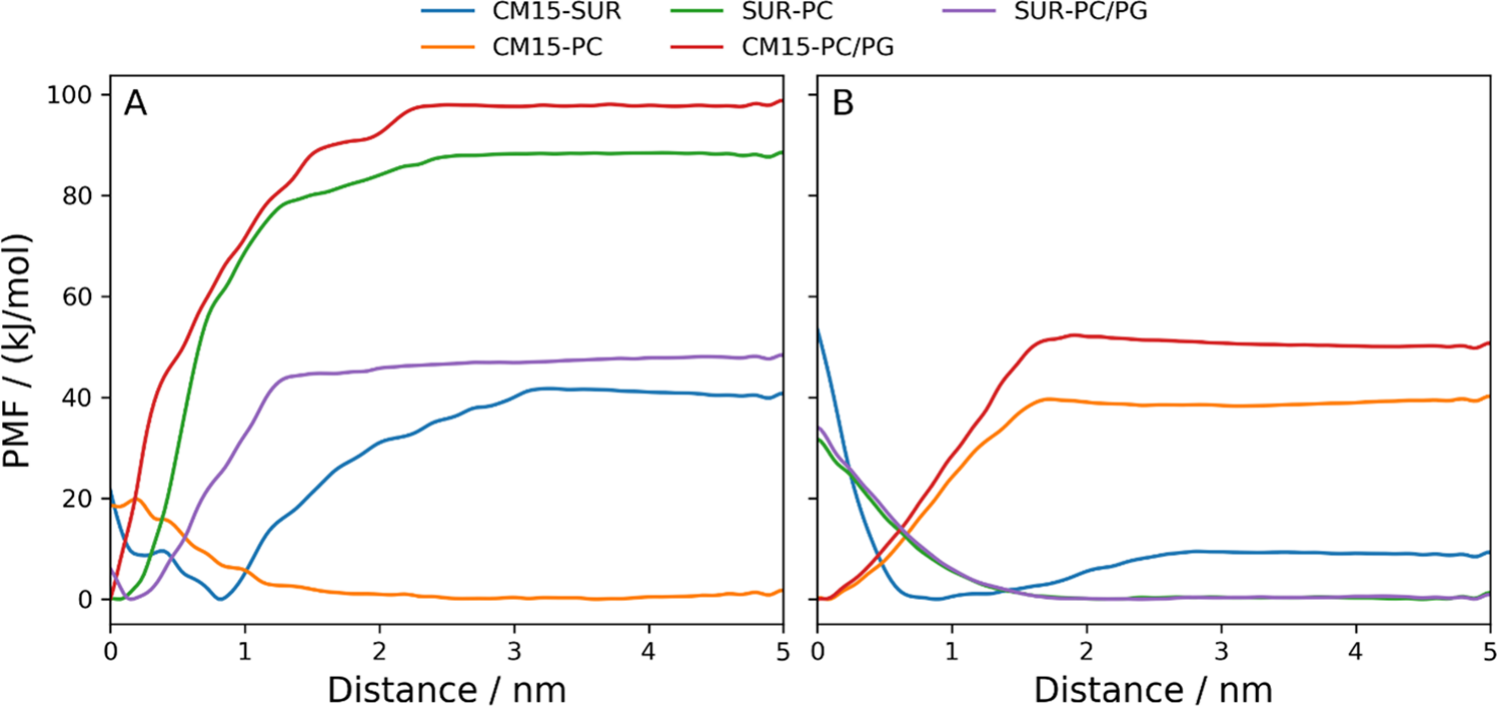
Potential of mean force (PMF) curves of the interacting components in (A) all-atom and (B) coarse-grained simulations. The collective variable was chosen to be the distance from the bilayer surface. In the case of the CM15–suramin interaction, it was chosen to be the distance between their COMs.

Our results have revealed several characteristics of the interactions between CM15, suramin, and bilayers mimicking different membrane environments. Some of those interactions, such as the interaction between suramin and CM15 or CM15 and the PC/PG bilayer well agreed between the all-atom and coarse-grained approaches, although others like the suramin–PC or CM15–PC interactions showed certain differences. Hence, to evaluate the discrepancies between the MD and CG calculations, and to assess in detail whether these results are in line with experimental findings, we performed a pairwise comparison of the investigated components in terms of free energy calculations using the umbrella sampling method (Figure 10).

As depicted, the above-described differences are very well reflected in the PMF curves. Therefore, in the following, the potential reasons for these discrepancies are discussed while taking into account the corresponding experimental results as well. For better clarity, the interactions are classified into three groups: those between the CM15 and the bilayers, the CM15 and suramin, and the suramin and the bilayers are discussed separately.

#### CM15–Bilayer Interactions

As described above, when comparing results obtained from the two computational approaches employed here, we noted some differences, which might be attributed to variations in free energy of the corresponding interactions; thus, free energy calculations were carried out on both all-atom MD and CG scales.

Regarding the peptide–bilayer interactions, the binding of CM15 to the PC bilayer was slightly unfavorable in all-atom simulations, while the peptide favored PC bilayer on the coarse-grained level. Nonetheless, this discrepancy has also been reported in previous computational studies. Bennett et al. have found in their CHARMM36 all-atom simulation on CM15 with PC bilayers that the peptide remained in the water phase for ∼1.7 μs when started from a disordered structure but the folded one bound easily when initiated near to the bilayer.^82^ Wang and his co-workers had also shown that CM15 binding to PC is heavily influenced by the conformation of the peptide, although, contrary to Bennett et al., their results indicated that peptide binding and insertion were reduced when CM15 adopted a prefolded α-helical initial structure.^81^ Coarse-grained studies on various positively charged antimicrobial peptides commonly indicated high affinity toward PC bilayers, even higher than expected based on the experimental results, which is also consistent with the present CG calculations.^83,84^ However, it is widely accepted that antimicrobial peptides, in general, show significantly higher affinity toward negatively charged bilayers, which selectivity was found in all-atom simulations.

When comparing the calculations to our previous experimental results, it can be seen that using circular dichroism (CD) spectroscopy, only a weak interaction was detected with neutral PC liposomes when monitoring peptide conformational changes upon lipid binding.^39^ Moreover, a relatively large variation can also be detected for experiments, exemplified by a more apparent interaction including peptide insertion into the lipid acyl-chain depth based on attenuated total reflection-Fourier transform infrared (ATR-FTIR) spectra for dry film samples or based on leakage assays where CM15 disrupted zwitterionic lipid vesicles despite that it is otherwise known for its low hemolytic activity.^44,85^ Notably, observations can also be affected by end terminal protection and employed experimental conditions.^86^

Thus, taking all of the previous computational knowledge and the available experimental results together, CM15 has some effect on PC bilayers, although a certain duality in the results can be identified. The all-atom and coarse-grained simulations demonstrated a similar duality despite the usage of state-of-the-art force fields. This suggests that an intermediate, weak interaction or structural dependency could be the effect, or other aspects take effect, which could be revealed neither with the experimental approaches nor with the currently applied all-atom and coarse-grained force fields. Most important, however, the differences between the water, PC, and PC/PG systems observed here are conceptually all in line with the experiments, including the presence of large aggregates for water and PC systems and the dissolution of these into PC/PG liposomes.

#### CM15–Suramin Interactions

The CM15–suramin interactions displayed the same characteristics for both approaches used, which is also supported by similarities in their free energy profiles (Figure 10). In the all-atom free energy calculation, the energy minimum around 0.8 nm between their COMs indicates that further compression of the molecules below this value is already unfavorable, potentially due to their high flexibility, as indicated by their high root-mean-square deviation (RMSD) values obtained for the solvent phase (Figure S26). Moreover, their flexible nature could also be the reason for the prevalence of aggregate formation over bilayer binding, as the effective interaction distance is higher for the former than that for the latter, which makes their interaction more probable in the relatively dense system. Therefore, the similarity between PMF curves obtained from both calculations (Figure 10A,B) confirms that the many-molecule aggregation processes characterized at the coarse-grained level can be considered as the simple analogous extension of the complex formation processes calculated for a reduced number of molecules at the all-atom level.

Our previous dynamic light scattering (DLS) and transmission electron microscopy (TEM)-based experimental results also suggested forming complex assemblies incorporating both suramin and CM15 molecules. Moreover, further experiments on the peptide–drug–membrane systems assessing the effect of one component on the others were also performed by varying the mixing order. It was demonstrated that preincubation of the CM15–suramin system and subsequent addition of lipid vesicles resulted in the largest aggregates. Nonetheless, less pronounced aggregation still occurred in those cases when the CM15 and suramin were not preincubated, or the mixing order was altered. It was also hypothesized that perturbations in the lipid head group region detected by IR spectroscopy and fluorescence quenching could be the result of the formation of dynamic complex associates between the suramin and the lipid-bound peptide.

#### Suramin–Bilayer Interactions

The results observed by simulations for the suramin–bilayer interactions indicated that suramin has the opposite propensity to interact with the bilayers in the all-atom vs the CG simulations (Figure 10). On the one side, this can be explained by parametrization issues of suramin, leading to the fact that the amphipathic nature of the suramin could not be captured in the parametrization process for a CG level, as was clearly outlined in the validation section (Section I.2, SI). On the other side, it can also be assumed that due to the rougher approximation of the particles and the fact that nonpolarizable MARTINI coarse-grained simulations have challenges to reproduce cation−π interactions,^87^ the lipid head group cholines have less freedom to compensate effectively for the electrostatic repulsion between the suramin sulfonates. These two reasons together can account for the rather opposite nature of the suramin–bilayer interactions in the two types of simulations.

**Figure 11:**
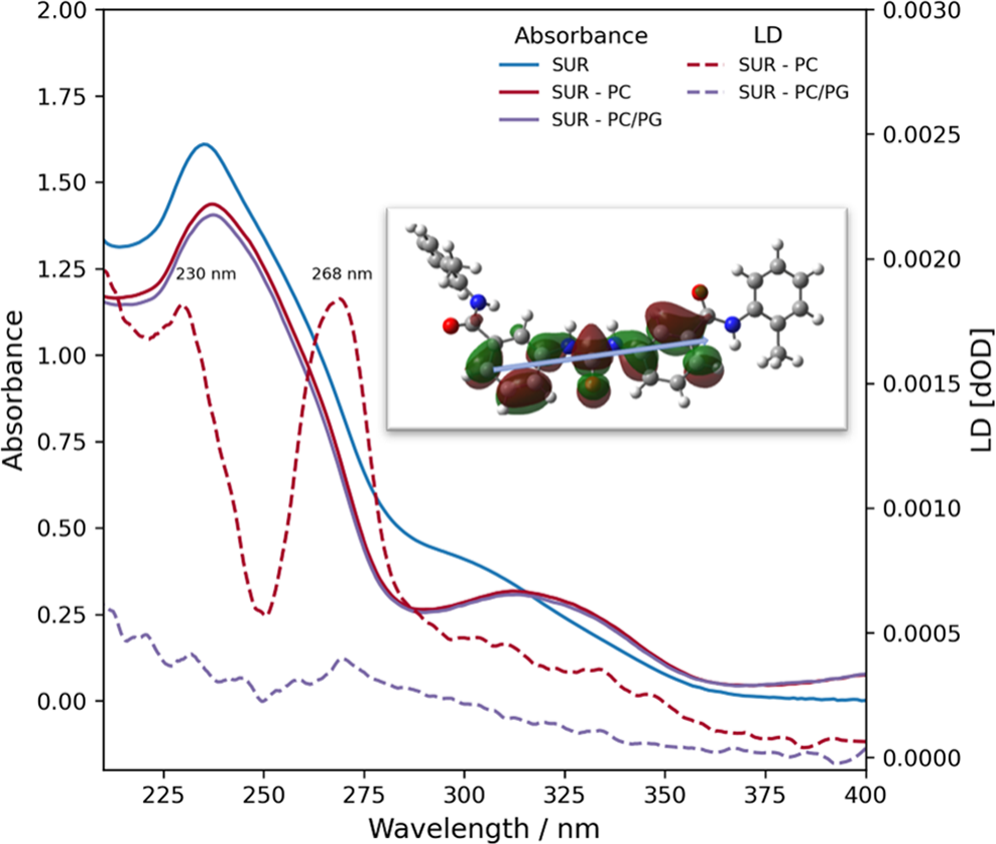
Lipid binding of suramin assessed experimentally. LD and UV–vis spectra of suramin in PC and PC/PG environments. The inset image shows an example of natural transition orbital (NTO) involved in the excited state at 239 nm with an oscillator strength *f* = 0.7565 obtained from QM calculations for the central part of suramin. The blue arrow shows the approximate direction of the transition dipole moment of the particular ground to an excited state.

To better put our calculations into context, we compared them with our previous and current experimental findings. The high affinity of suramin to bind to the surface regions of neutral lipid bilayers observed in all-atom simulations gives a reasonable explanation for the suramin-induced perturbations in lipid phosphate vibrations detected by ATR-FTIR spectroscopy.^39^ To further validate our MD simulations on the orientation of bilayer-bound suramin, the experimental proof was obtained from LD spectroscopic data supported by quantum chemical (QM) calculations as well (Figure 11).

According to the theory of LD spectroscopy, LD signals can arise only from oriented compounds; therefore, the intense positive peaks with PC liposomes at 230 and 268 nm in the LD spectrum are a clear indication of bilayer-bound suramin. Moreover, the positive sign of these peaks means that the electric transition dipole moment (TDM) vectors of the corresponding excited states of suramin are oriented parallel to the orientation direction. For identifying the orientation of the corresponding transition dipole moment, we have performed quantum chemical calculations on suramin (for details, see the Methods section). Previous calculations aiming to assign TDMs to corresponding peaks observable in LD spectra^88−90^ have demonstrated that the low-energy conformer of the most extended conjugated system within a particular molecule gives rise to the main TDMs. Considering the structure of suramin, it can be seen that its central part, a urea moiety that has a significant π-conjugation in a near planar form, together with the adjacent two phenyl rings, will together comprise the main conjugated area of the molecule. This part is not influenced significantly by the additional aromatic rings as these are separated by peptide bonds from the central core. The quantum chemical calculations have shown that the TDMs corresponding to the LD peaks are parallel to the long axis of the suramin molecule (Figure 11 and Table S2). As the orientation direction is perpendicular to the membrane normal, it follows that the long axis of the suramin molecule is also perpendicular to the membrane normal. These results suggest drug–membrane interaction, where the central part of the small molecule occupies a well-defined position with the aromatic rings oriented mainly parallel relative to the bilayer surface, which is in line with the results of the all-atom simulations of suramin with the PC bilayer (Figure 3B). In the presence of PC/PG, suramin LD peaks could still be detected, however, with much weaker intensity, in concert with computational results suggesting lower suramin affinity toward charged PG lipids.

Based on all of the above considerations, the two theoretical techniques together give an insight into the nature of CM15–PC bilayer interactions and the lower affinity of CM15 towards zwitterionic bilayers, particularly when compared to the PC/PG bilayer. It should be noted, though, that similarly to the employed theoretical techniques, the experimental results regarding the CM15–PC interactions can also be ambiguous and provide somewhat contradicting results concerning the affinity of CM15 toward PC bilayers. Most likely, this is due to the low relative free energy differences between various states of the system, and thus, the employed technique or environment can have a decisive effect on its behavior.

Addressing CM15–suramin binding affinity and aggregate formation, the general conclusions of all-atom and coarse-grained simulation results were also in line with the ones obtained by experimental measurements. The higher tendency to form CM15–SUR complexes in the presence of pure PC over PC/PG also demonstrated experimentally^39^ was seen in the coarse-grained simulations. Note that a direct comparison between experiments and simulations is always challenging, as in the present case, the latter approach allowed the study of these systems in a more competitive way where each component has the chance to find its preferred binding partner, according to the order of their affinity, rather than in experiments where the mixing order of a three-component system creates somewhat predefined conditions. In summary, the simulations also confirmed that peptide–suramin complexes could bind to the lipid bilayer, revealing which component might face the lipids to facilitate the interaction as indicated in the all-atom approach for the 1:1 peptide–suramin system (Figure 3).

## Conclusions

In the present computational study, we have analyzed the interactions between a model antimicrobial peptide, CM15, and a polyanionic, small-molecule drug, suramin, and model lipid bilayers mimicking mammalian and bacterial cell membranes, with a particular focus on their ability to form aggregates.

In good agreement with previous experimental findings, we have found that the peptides and drug molecules quickly form semistructured associates, but this aggregation tendency is highly influenced by bilayer composition in a complex competing interaction network. Consistent with the experimental measurements, our all-atom calculations indicated that suramin could bind to neutral membrane components, and this is dictated by its hydrophobic central part facing the hydrophobic interior of the lipid and the cation−π interactions between the lipid choline head groups and the drug naphthyl moieties. Although the bacterial membrane mimicking the PC/PG model bilayer enhances CM15 affinity and suppresses suramin binding, the formed peptide–drug aggregates could still bind to both bilayers. Therefore, suramin could reduce the overall selectivity of AMPs by allowing an alternative binding mode to nonspecific neutral bilayers and potentially also decrease their activity due to the reduced conformational flexibility in the formed aggregates. We have also carried out microsecond-scale coarse-grained simulations, for which we parametrized suramin. The results have shown that the formation of peptide–drug aggregates is a collision-driven process where dissociation of components could also occur. It was also demonstrated that coarse-graining and simulating peptide–small-molecule systems could give valuable insights into the underlying molecular mechanism of their interaction. Although discrepancies in some of the interactions were found in coarse-grained simulations compared to their all-atom counterparts, the results balanced from the two computational methods correlate well with our previous and new experimental findings. Therefore, the computational approach used here might serve as a mechanistic tool to investigate the interaction network in similar three-component systems where peptide–drug complex formation leads to aggregation, as observed for several examples in our laboratory and also by others.

Most importantly, the simulations on three-component systems have demonstrated that in water, the CM15–suramin assemblies form readily into a large aggregate; however, when a PC bilayer is present, the aggregates become somewhat smaller with some individual peptides entering the bilayer head group region. Finally, when considering PC/PG bilayers, the CM15–suramin associates do not build up, rather small initial complexes all “dissolve” in the bilayer surface regions. Although one particular system was analyzed, the general aspects of this system may provide an outlook with far-reaching consequences. Recent experimental results, employing various peptides and small molecules such as food colors, endogenous metabolites, or bacterial siderophores, together with current simulations, suggest that AMPs are often present as associates, potentially with counter-charged compounds, in in vivo systems. Thus, a likely scenario is that when meeting host cell membranes, these associates may be affected, but to a lesser extent, potentially leading to limited toxic function on neutral bilayers. However, when they are in the proximity of a negatively charged, i.e., microbial membrane, the associate formation could be turned backward, and AMPs could start to enter into the bilayer surface regions in a monomeric or single drug-peptide complex form, exerting membrane toxicity on the target organism. While understanding this mechanism will require further studies, the fact that certain AMPs can be directed into semistructured assemblies, which have a different affinity toward neutral and negatively charged membranes, can provide an alternate approach for pharmaceutical developments, where peptides are targeted by drugs in a rational manner.

## Supporting Information Available

Description of methods applied to parametrize suramin for MARTINI force field; additional figures depicting parameter distribution overlaps after parametrization; kernel density distributions of the umbrella sampling windows; figure showing the DOPC–choline vs suramin–naphthalene distances; additional figures showing CM15 and suramin distances of the 4:4 CM15–suramin system in different environments; figure indicating the result of the RMSD analysis of suramin in aqueous phase; and table summarizing the excitation energies, wavelengths, and oscillator strengths of the QM calculations (PDF)
