# Supplementary material for: Controlling Peptide Function by Directed Assembly Formation: Mechanistic Insights Using Multiscale Modeling on an Antimicrobial Peptide–Drug–Membrane System
Source: ACS Omega. 2021 Jun 11;6(24):15756–69. doi: 10.1021/acsomega.1c01114 (PMC8223213; doi:10.1021/acsomega.1c01114)
Supplement: Supplementary file 1 — ao1c01114_si_001.pdf [file ao1c01114_si_001.pdf]

Supporting Information:

CONTROLLING PEPTIDE FUNCTION BY DIRECTED ASSEMBLY FORMATION: MECHANISTIC INSIGHT USING MULTISCALE MODELLING ON AN ANTIMICROBIAL PEPTIDE – DRUG – MEMBRANE SYSTEM

*Gergely Kohut[1,2], Tünde Juhász[1], Mayra Quemé-Peña[1,2], Szilvia Erika Bősze[3], Tamás Beke-Somfai[1]**

[1]Institute of Materials and Environmental Chemistry, Research Centre for Natural Sciences, Magyar tudósok körútja 2, H-1117 Budapest, Hungary

[2]Hevesy György PhD School of Chemistry, ELTE Eötvös Loránd University, Pázmány Péter sétány 1/A, H-1117 Budapest, Hungary

[3]ELKH Research Group of Peptide Chemistry, Eötvös Loránd University, Pázmány Péter sétány 1/A, H-1117 Budapest, Hungary

I. Extended Methods

I.1. All-atom simulation protocols

Table S1. Applied restraints during the minimization and equilibration process of the all-atom simulations.

Restraints	k / (kJ mol^{-1} nm^{-2})						
	STEP0	STEP1	STEP2	STEP3	STEP4	STEP5	STEP6
suramin heavy atom positions	1000	1000	1000	400	200	40	0
CM15 backbone heavy atom positions	1000	1000	1000	400	200	40	0
CM15 sidechain heavy atom positions	1000	1000	400	200	40	0	0
Lipid phosphorous atom positions	1000	1000	1000	400	200	40	0
Lipid C3-C1-C2-O21 dihedral	1000	1000	400	200	200	100	0

I.2. Suramin parametrization and validation protocols

Parametrization of Suramin. The suramin molecule was parametrized using the beta version of the MARTINI v3.0[1] force field. It was used instead of the latest MARTINI v2.2[2] due to its better performance during parameter validation when applied to the mapping.

The all-atom reference data set was generated using the CHARMM36m[3] force field with the same simulation as for the other all-atom simulations. Suramin was simulated in the water phase with TIP3P water molecules, it was neutralized by Na^+ atoms, and the NaCl salt concentration was set to 150mM. The equilibration process was started by the steepest descent minimization in 5000 steps, which was followed by two restrained 25 ps long NVT simulations with Berendsen and velocity rescale coupling. Next, 450 ps NPT type equilibration was performed in three steps with Berendsen pressure coupling but different restraints on the suramin heavy atoms. As a final step of the equilibration, a 100 ns simulation was carried out with 200 kJ mol^{-1} nm^{-2} restraints on suramin heavy atoms using Nosé-Hoover thermostat with 1.0 ps coupling time and an isotropic Parrinello-Rahman barostat with 5 ps coupling to ensure the correct statistical ensemble. Finally, the 50 ns unrestrained production run was performed with the same parameters as in the last equilibration step. The step size was 2 fs, and the atomistic coordinates were written out in every ps, which resulted in 50000 frames for data fitting and validation. The coarse-grained representation of the molecule was created by an atom-to-bead mapping developed here (**Figure 2**, main text).

Based on the mapping scheme, the atom-to-bead mapped trajectory was created, and the initial bond, angle, and dihedral information was extracted and averaged over the whole simulation. The averaged values were then used as initial parameters for coarse-grained suramin. After building the initial structure, it was solvated with water molecules and neutralized by Na^+ ions where needed. The system was first simulated for one ns as an NVT ensemble (τ_t = 1.0 ps) coupled to a velocity rescaling thermostat with a target temperature of 300 K, which was followed by a 50 ns NPT simulation in 1 bar, controlled by an isotropic Berendsen barostat (τ_p = 6.0 ps, κ_p= 4.5 10^{-4} bar^{-1}). The trajectory was then analyzed and compared to the all-atom simulation results in terms of the bond, angle, and dihedral distributions, and an iterative process was applied to optimize the initial parameters using Python and R scripts.

Next, the production run was performed for 1 μs as an NPT ensemble, and velocity rescale thermostat was used for temperature coupling (τ_t = 1.0 ps) and Parrinello-Rahman barostat for pressure coupling (τ_p = 16.0 ps, κ_p= 3.0 10^{-4} bar^{-1}). The step size for every CG simulation was 20 fs.

The parameter validation process took place in two steps. First, the CG bond, angle, and dihedral parameters were compared directly, while in the second step, inherited descriptors such as the radius of gyration and octanol-water solvation free energy difference was matched with their all-atom counterparts.

The octanol-water solvation free energy difference was calculated using umbrella sampling[4] simulations and Plumed 2.4.3 software[5]. An octanol-water biphasic system was created where the suramin was in the center of the water phase neutralized by Na^+ atoms. The equilibration processes both for all-atom and CG simulations were the same as above. The collective variable of the umbrella sampling simulations was defined as follows:

$$d_z^{sur} = \left| \frac{r_z^{oct} - r_z^{sur}}{r_z^{oct} - r_z^{wat}} \right|$$

where r_z^x is the z coordinate of the center of mass (COM) of the corresponding phase or molecule. Consequently, when $d_z^{sur} = 1$, suramin is in the center of the water phase, when $d_z^{sur} = 0$, it is inside the octanol phase. The following umbrella potential was used:

$$U(d_z^{sur}) = \frac{\kappa}{2}(d_z^{sur} - d_z^{ref})^2$$

where the force constant, □ = 5000 kJ/mol, and d_z^{ref} is the reference position of the window. The selected 0.04 step size resulted in altogether 26 umbrella windows. Each umbrella was simulated for 25 ns, the coordinates were saved every 25 steps, and the last 10 ns were analyzed. The Potential of Mean Force was calculated using the variational free energy profile (vFEP) approach[6] at 300 K temperature.

Validation of suramin parameters. The evaluation of suramin in a CG setup is described and briefly compared with its performance in related MD calculations, emphasizing its relationship with the phase surface present between a hydrophobic and a hydrophilic environment. The solvent phase all-atom simulations were used as a basis for the suramin parametrization. As the mapped beads in the all-atom simulation defined narrow distributions in bond lengths and angles, the final bond, angle, and dihedral angle parameters were chosen accordingly. Consequently, most of the bonds were constrained, and stiff force constants were applied for the angles and for those bonds which were not constrained. Dihedral angles were defined to ensure the planarity of the sulfonated naphthyl rings, and the correct rotation of the two distinct parts of the suramin around the urea core. The distributions of the final parameters showed good overlap with their all-atom counterparts (Figure S2-S25, end of this section).

Besides the direct comparison of the distributions of the parameters between the CHARMM36 all-atom and the MARTINI v3.0b coarse-grained simulations, the parameters were also validated indirectly through two characteristics, the radius of gyration and the free energy difference of the water-to-octanol phase transition (**Figure S1**).

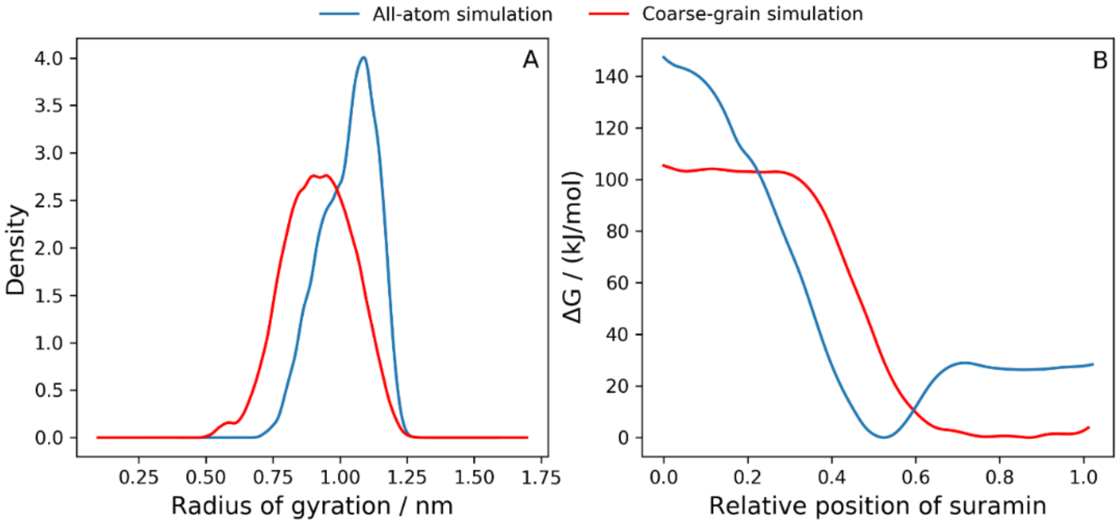

Figure S1. Results of the indirect parameter validations for suramin. (A) The distribution of the radius of gyration. (B) The potential of mean force (PMF) of the suramin when moved from the COM of the water phase (1.0) to the COM of the octanol phase (0.0).

There is a significant overlap between the two distributions in terms of the radius of gyration; however, the expected value is lower in the coarse-grained simulation, which suggests a slightly more

compact representation of suramin (**Figure S1A**). While the radius of gyration is a good structural descriptor, it cannot measure the correctness of the chosen bead types; hence we calculated the free energy difference of the water-to-octanol phase transition, which reflects better on the merit of the electrostatic and van der Waals interactions. We chose the water-octanol system as it is commonly used for parametrization by calculating partitioning free energies[2]. The PMFs show approximately the same free energy difference for the transition in both cases, with one remarkable exception (**Figure S1B**). Namely, the PMF of the transition in the CG simulation is a monotonic function, while it has a minimum at the phase boundary when simulated on an all-atom level. This indicates the “amphipathic” nature of suramin, which the coarse-grained parametrization could not capture. Nevertheless, the coarse-grained simulations show reasonable correspondence with their all-atom counterparts allowing its use in further simulations.

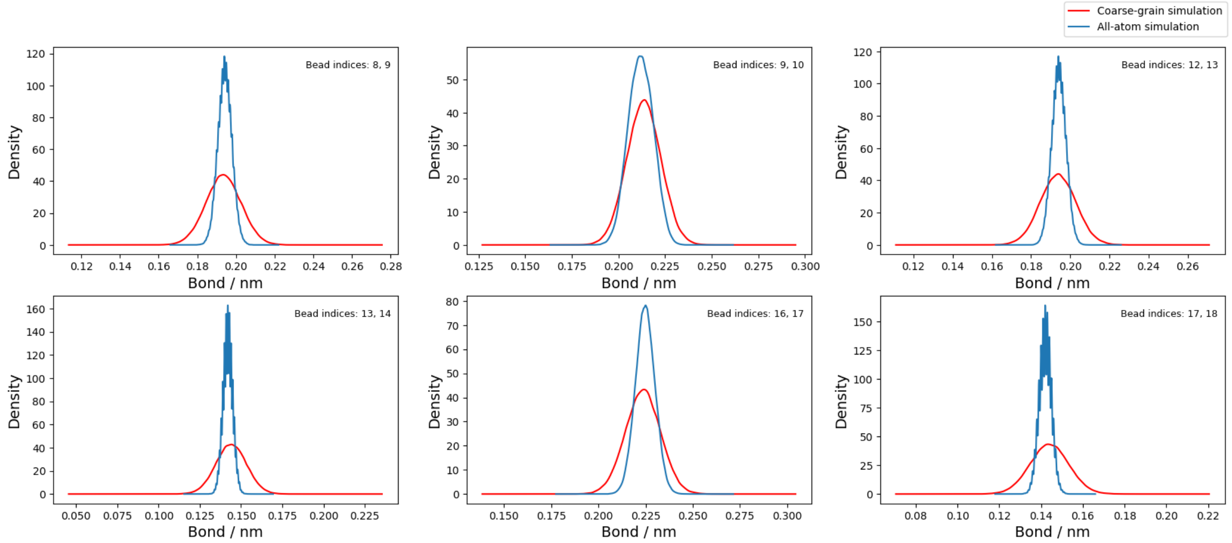

Figure S2. Kernel density distributions of suramin bonds of coarse-grained and all-atom simulations. Part 1/7.

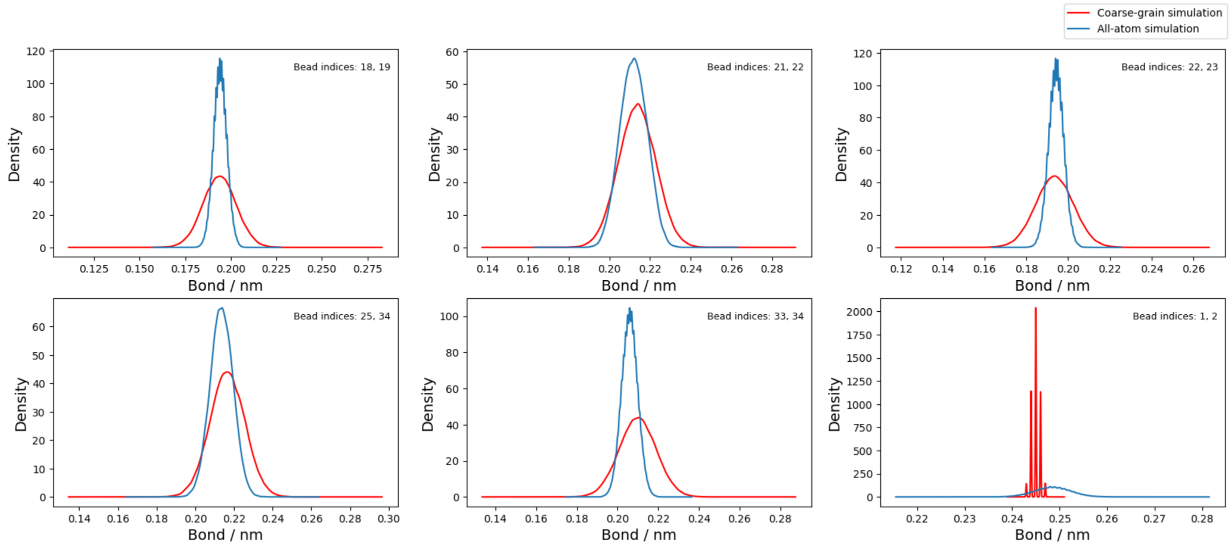

Figure S3. Kernel density distributions of suramin bonds of coarse-grained and all-atom simulations. Part 2/7.

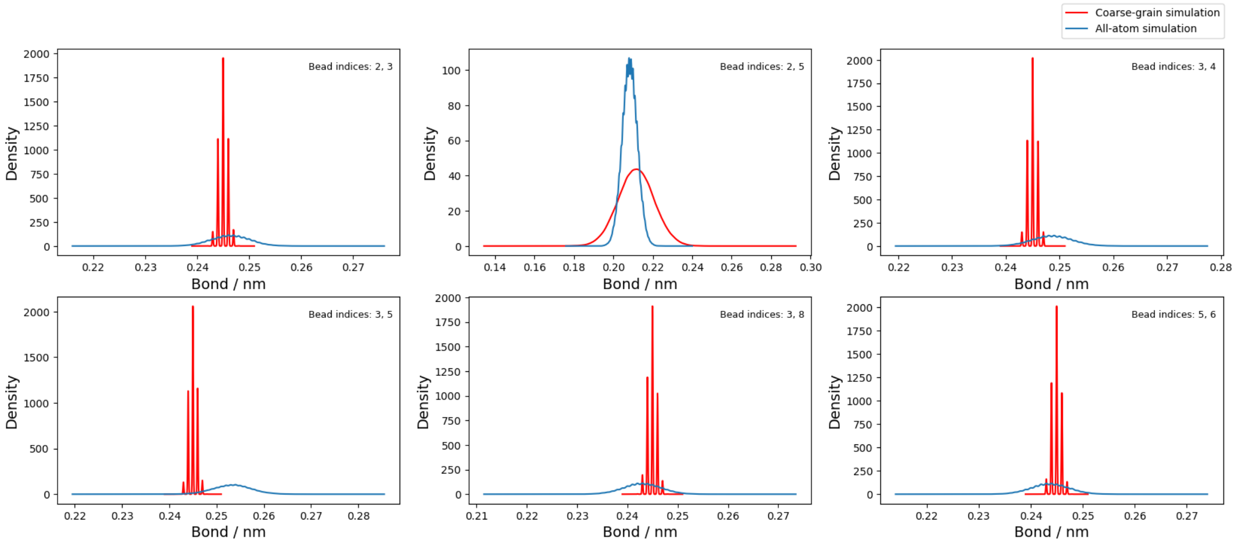

Figure S4. Kernel density distributions of suramin bonds of coarse-grained and all-atom simulations. Part 3/7.

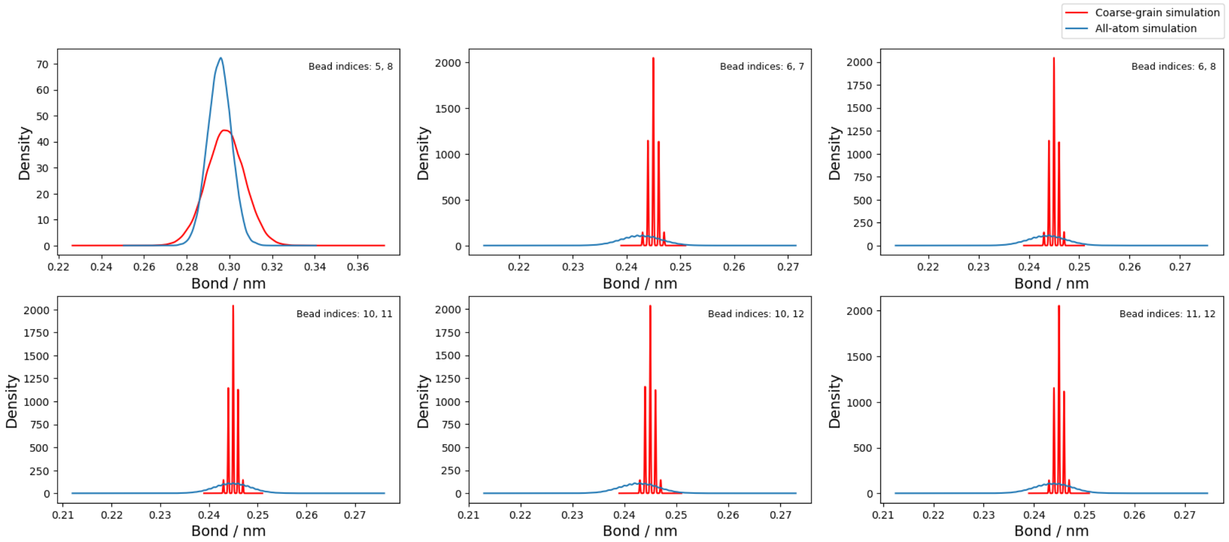

Figure S5. Kernel density distributions of suramin bonds of coarse-grained and all-atom simulations. Part 4/7.

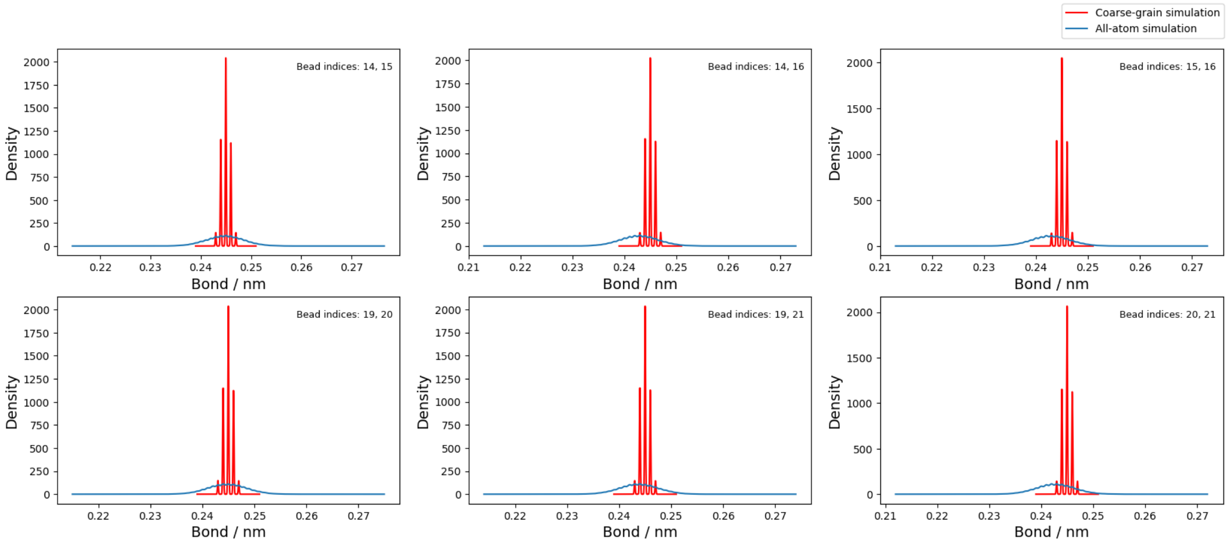

Figure S6. Kernel density distributions of suramin bonds of coarse-grained and all-atom simulations. Part 5/7.

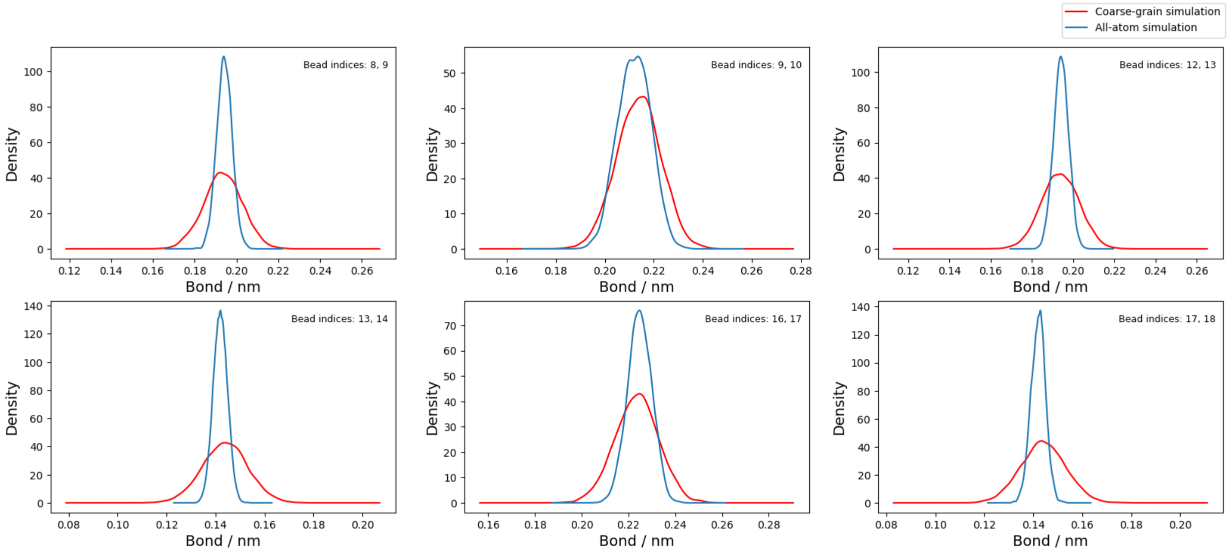

Figure S7. Kernel density distributions of suramin bonds of coarse-grained and all-atom simulations. Part 6/7

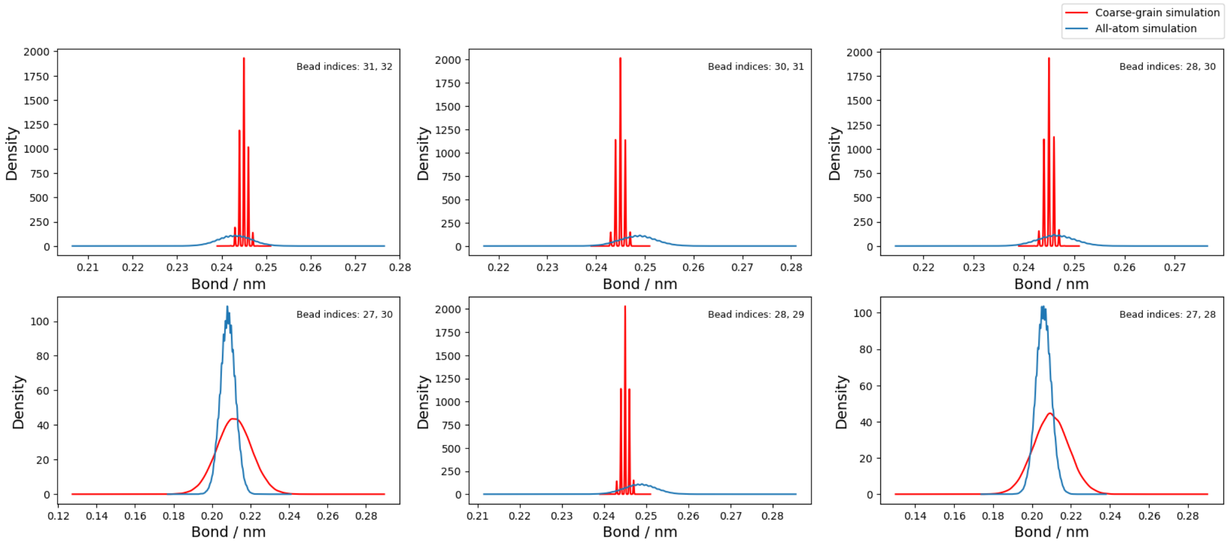

Figure S8. Kernel density distributions of suramin bonds of coarse-grained and all-atom simulations. Part 7/7.

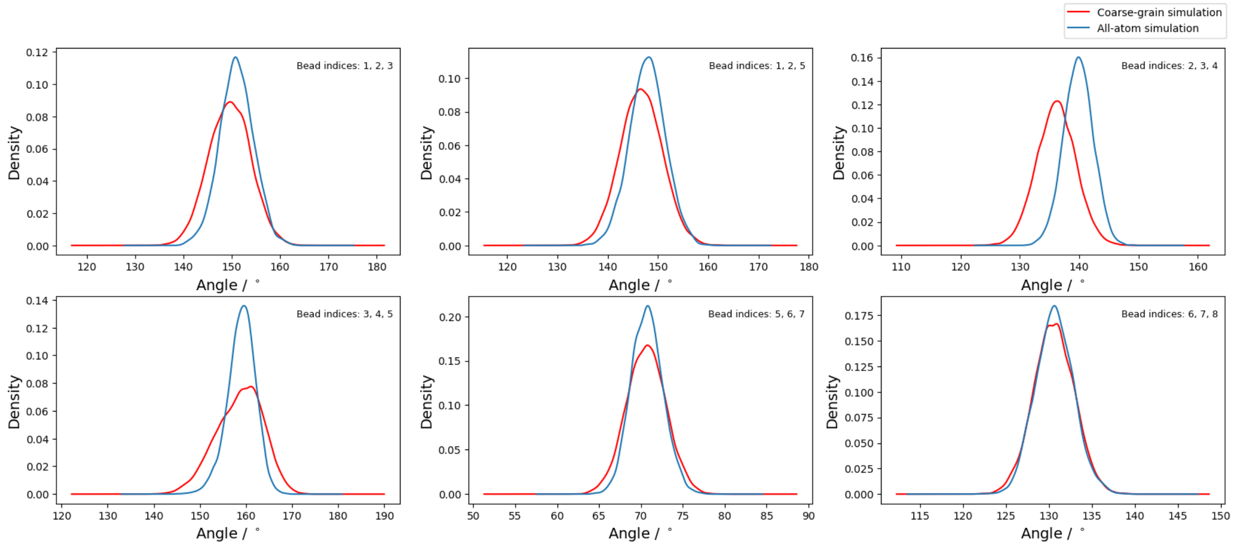

Figure S9. Kernel density distributions of suramin angles of coarse-grained and all-atom simulations. Part 1/7.

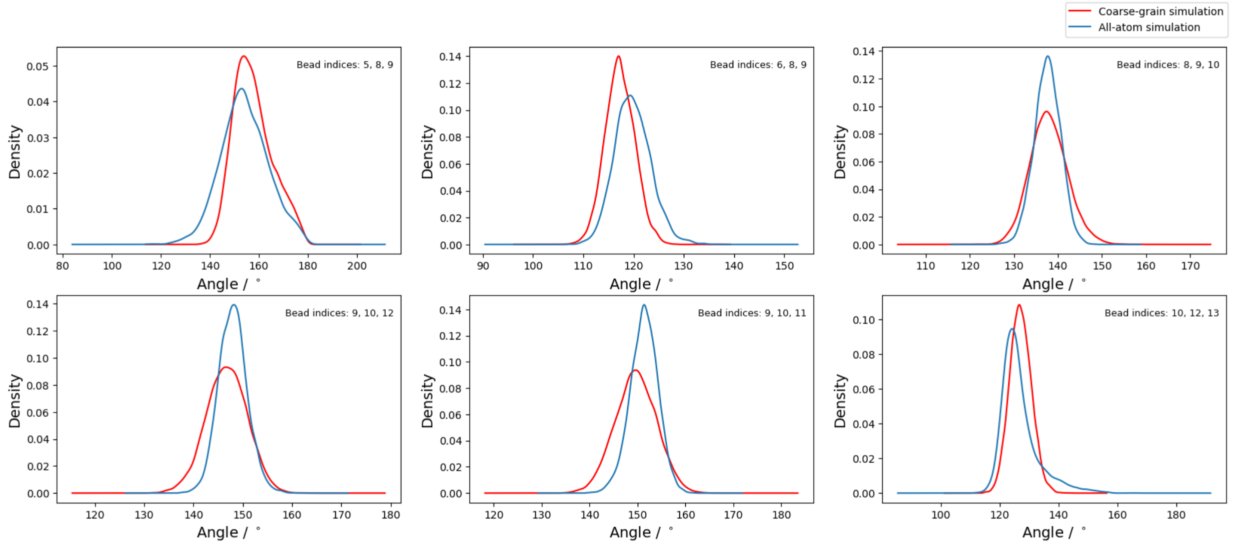

Figure S10. Kernel density distributions of suramin angles of coarse-grained and all-atom simulations. Part 2/7.

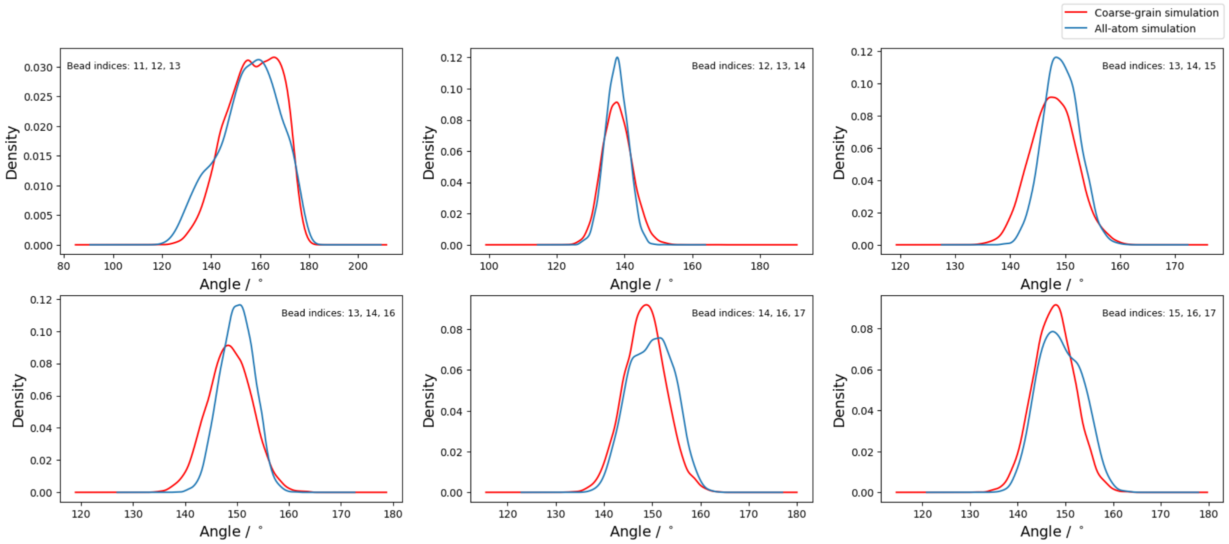

Figure S11. Kernel density distributions of suramin angles of coarse-grained and all-atom simulations. Part 3/7.

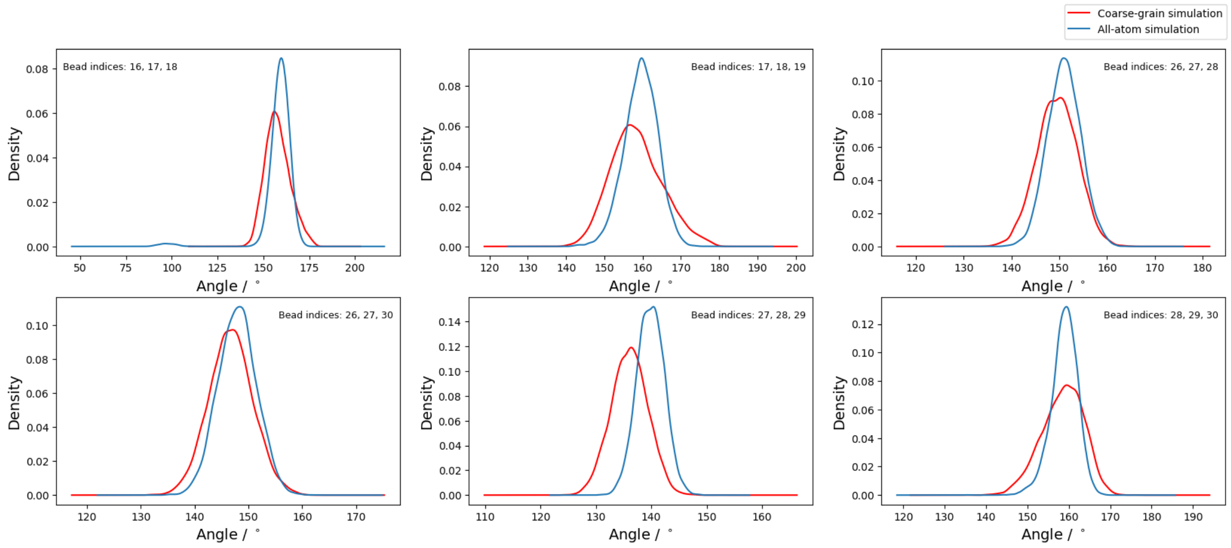

Figure S12. Kernel density distributions of suramin angles of coarse-grained and all-atom simulations. Part 4/7.

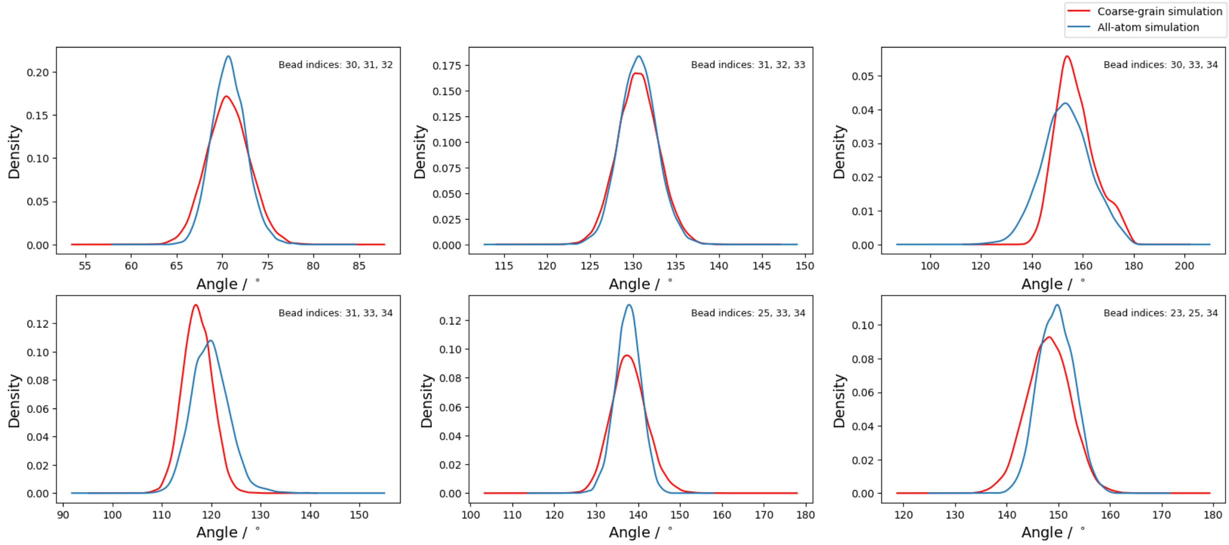

Figure S13. Kernel density distributions of suramin angles of coarse-grained and all-atom simulations. Part 5/7.

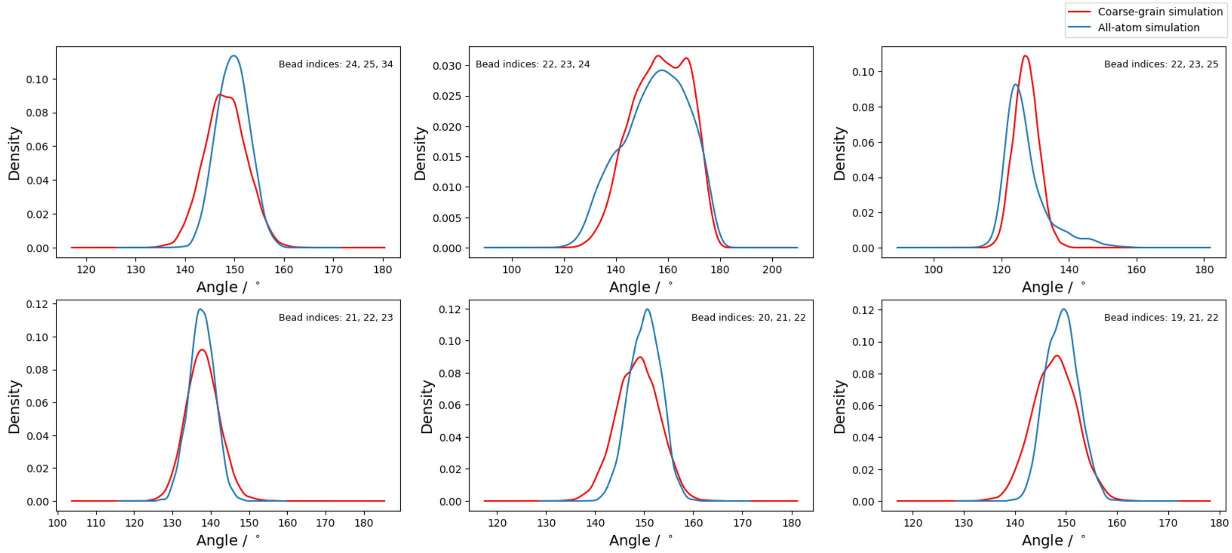

Figure S14. Kernel density distributions of suramin angles of coarse-grained and all-atom simulations. Part 6/7.

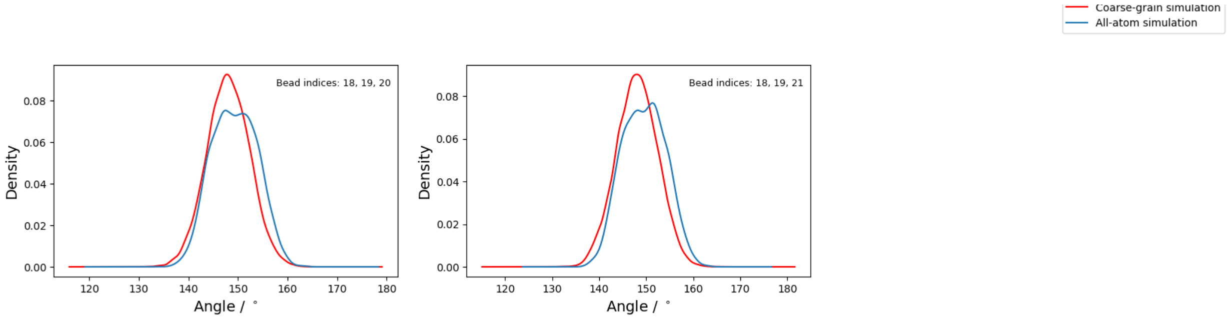

Figure S15. Kernel density distributions of suramin angles of coarse-grained and all-atom simulations. Part 7/7.

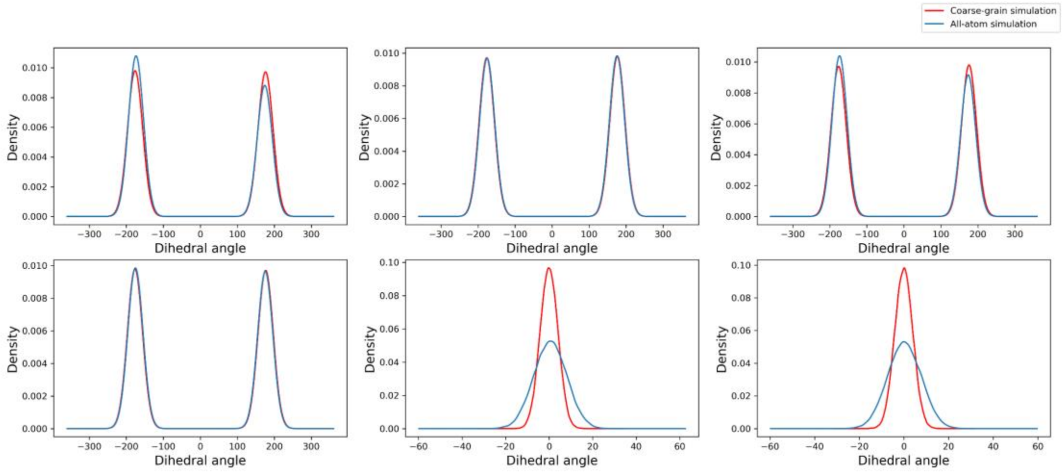

Figure S16. Kernel density distributions of suramin dihedral angles of coarse-grained and all-atom simulations.

I.3. Free energy calculations

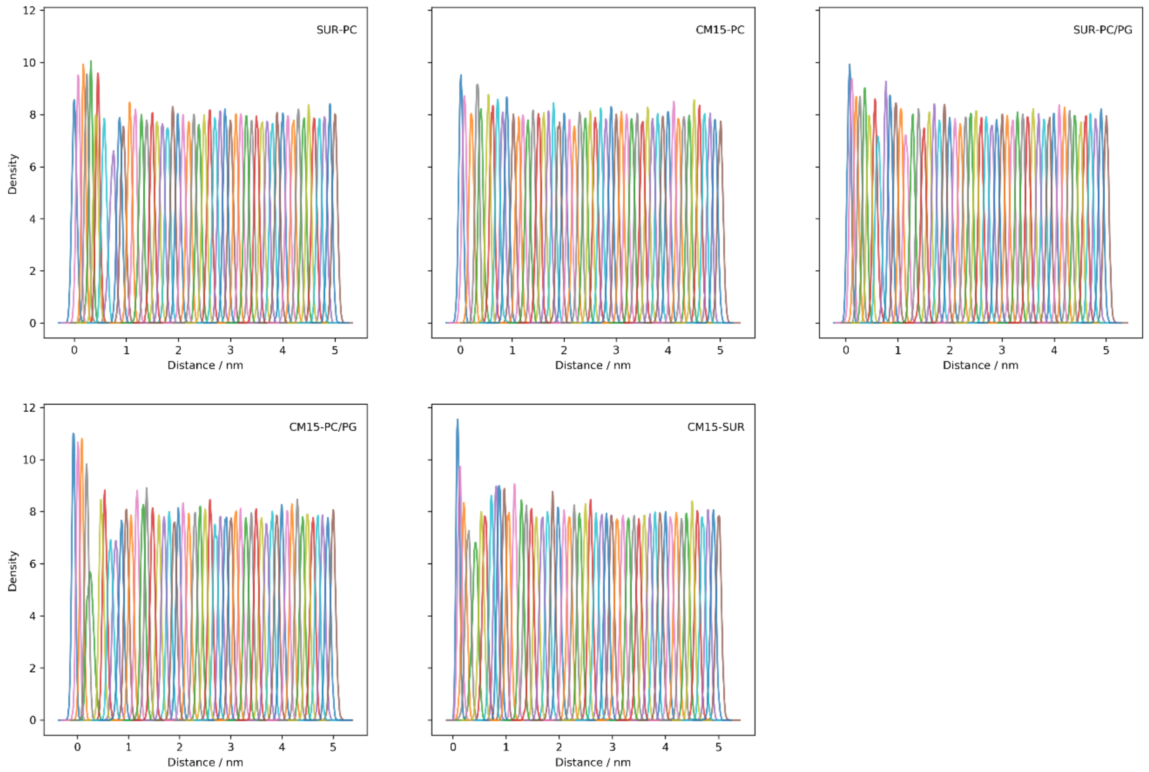

S17. Figure. Kernel density distributions of the distances of the US windows in the cases of the different all-atom simulations

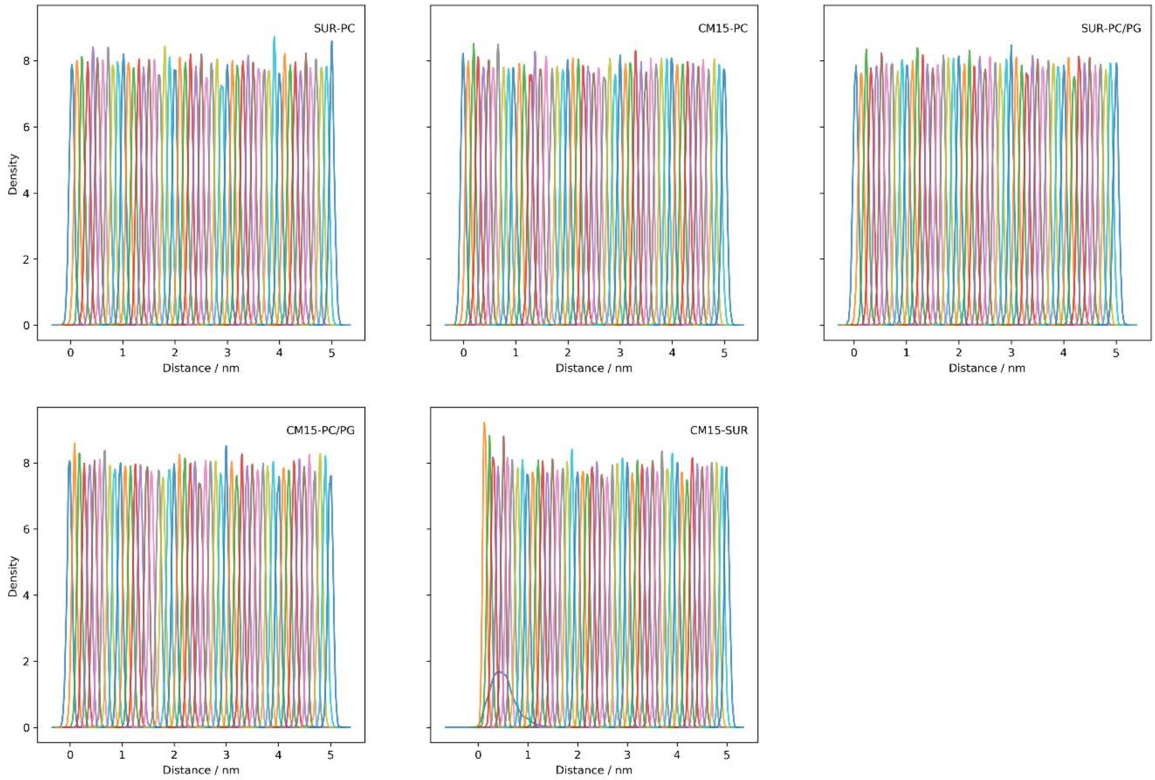

S18. Figure. Kernel density distributions of the distances of the US windows in the cases of the different coarse-grained simulations.

II. Extended Results

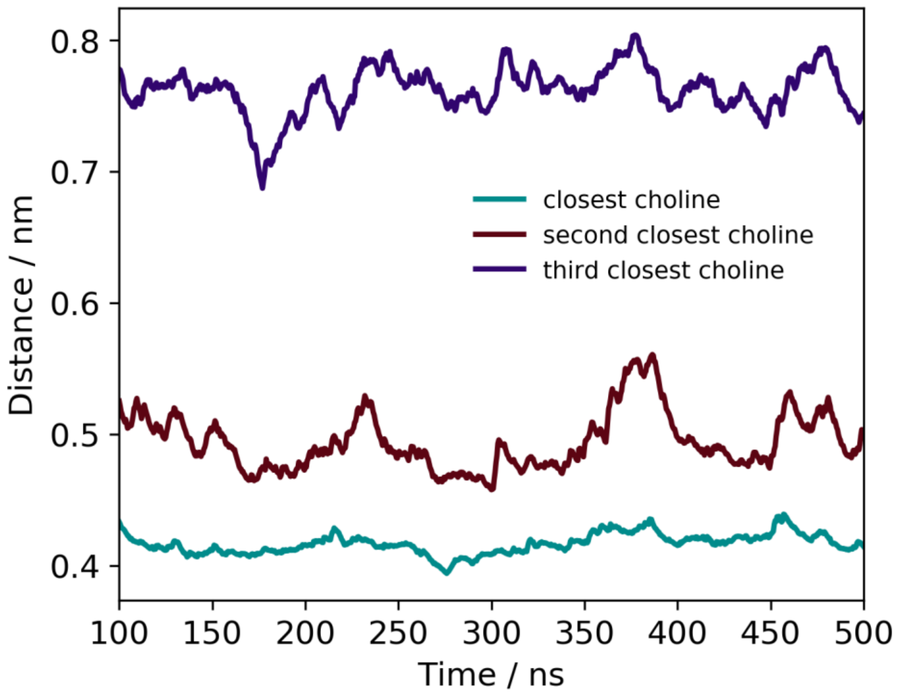

Figure S19. DOPC-choline vs suramin-naphtaline distances. The time dependence of the average distance between suramin's naphtaline rings and the closest (blue), the second closest (red) and the third closest (purple) choline are shown.

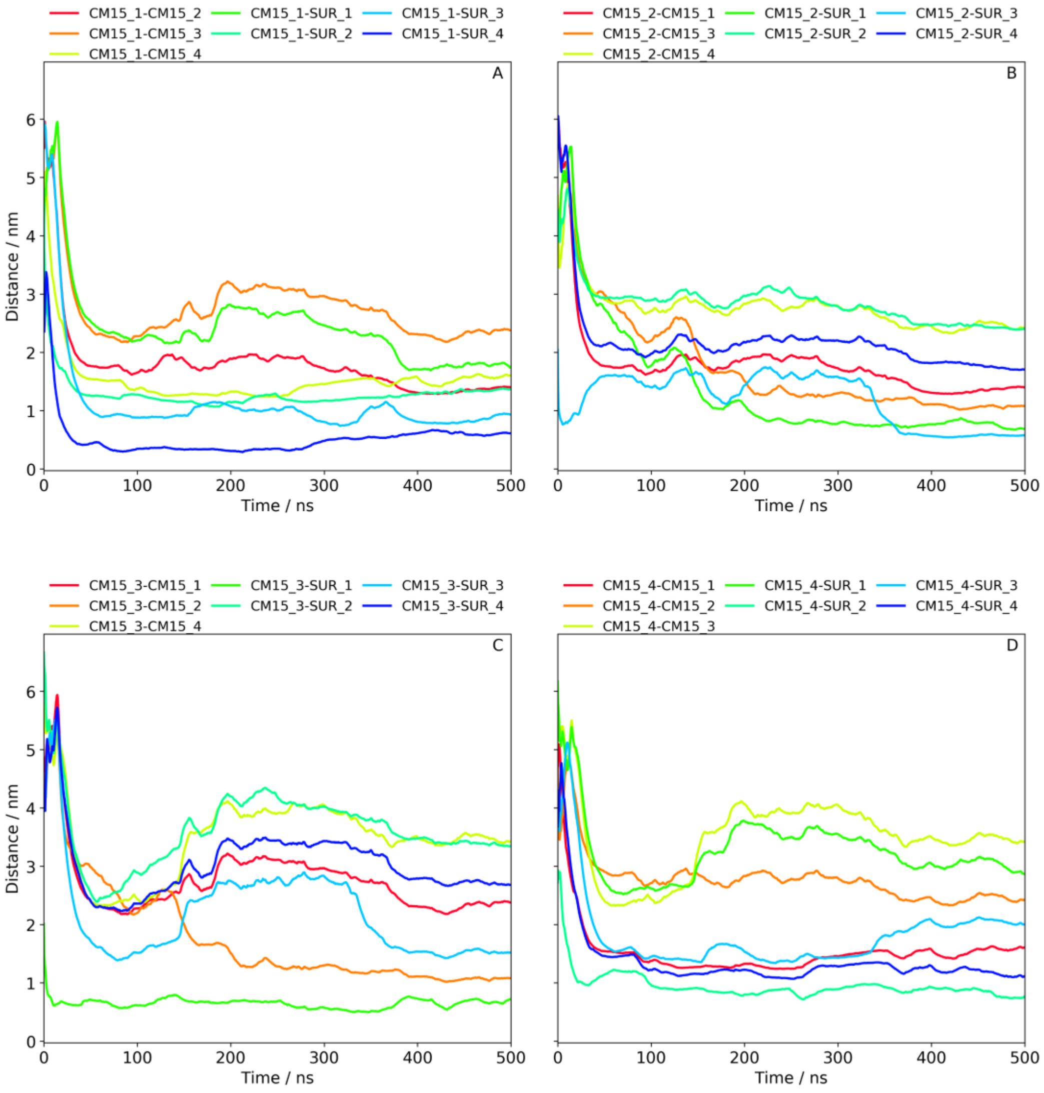

Figure S20. *CM15 distances* of the 4:4 CM15-suramin systems *in aqueous environment*. The distances between the first (A), second (B), third (C) and fourth (D) CM15 molecule and all other component are shown.

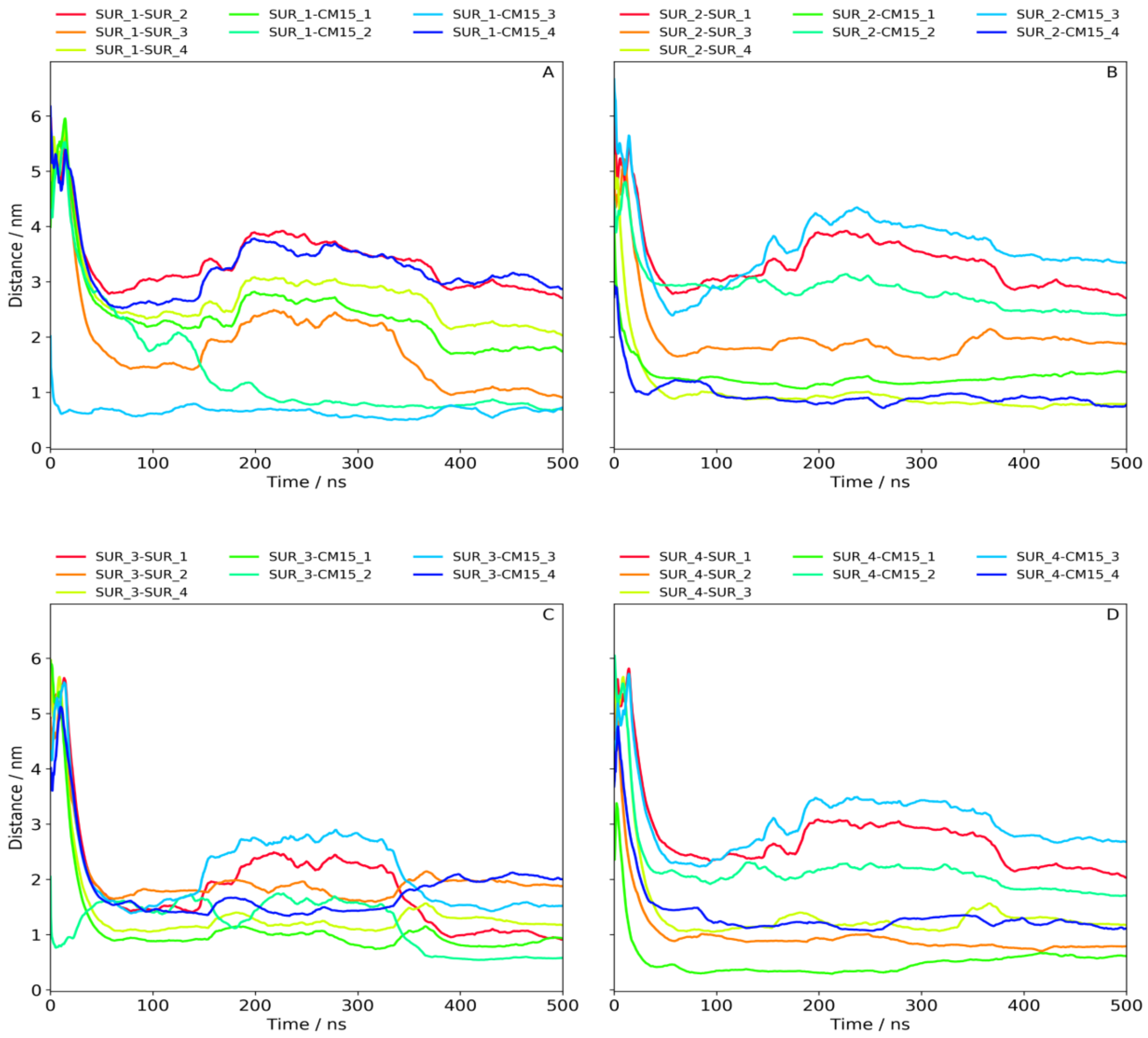

Figure S21. *Suramin distances* of the 4:4 CM15-suramin systems *in aqueous environment*. The distances between the first (A), second (B), third (C) and fourth (D) suramin molecule and all other component are shown.

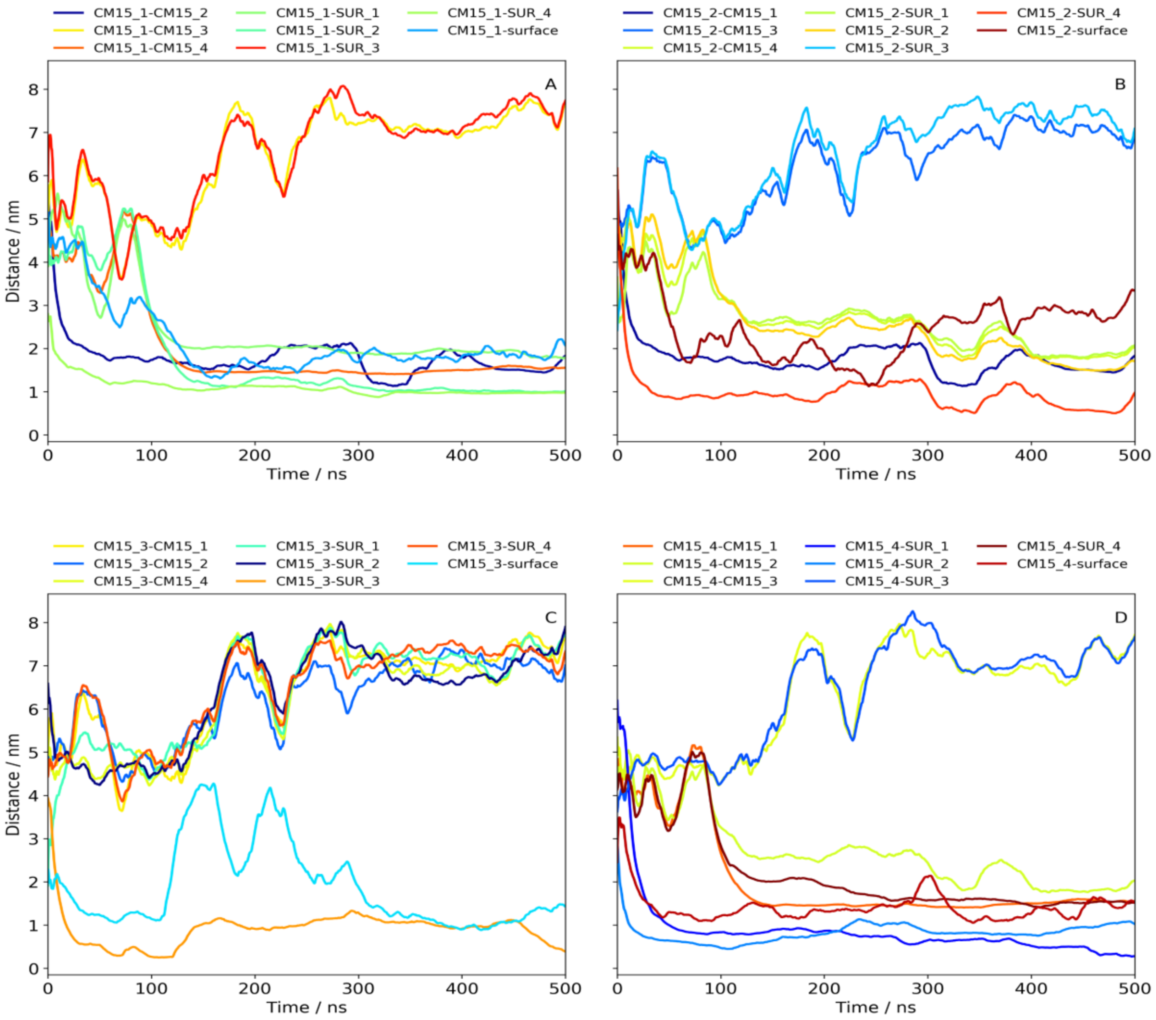

Figure S22. *CM15 distances* of the 4:4 CM15-suramin systems *in DOPC environment*. The distances between the first (A), second (B), third (C) and fourth (D) CM15 molecule and all other component are shown.

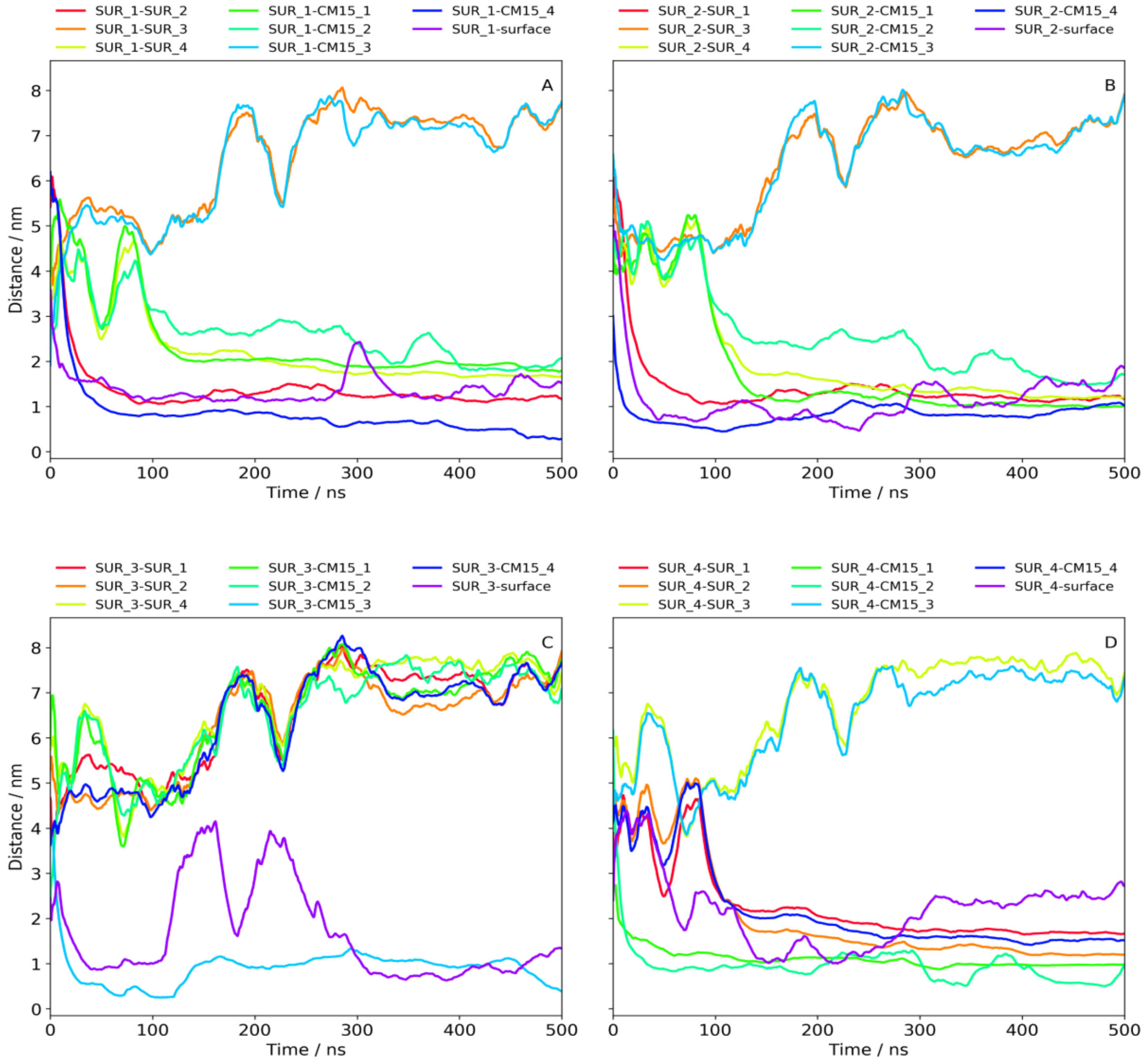

Figure S23. *Suramin distances* of the 4:4 CM15-suramin systems *in DOPC environment.* The distances between the first (A), second (B), third (C) and fourth (D) suramin molecule and all other component are shown.

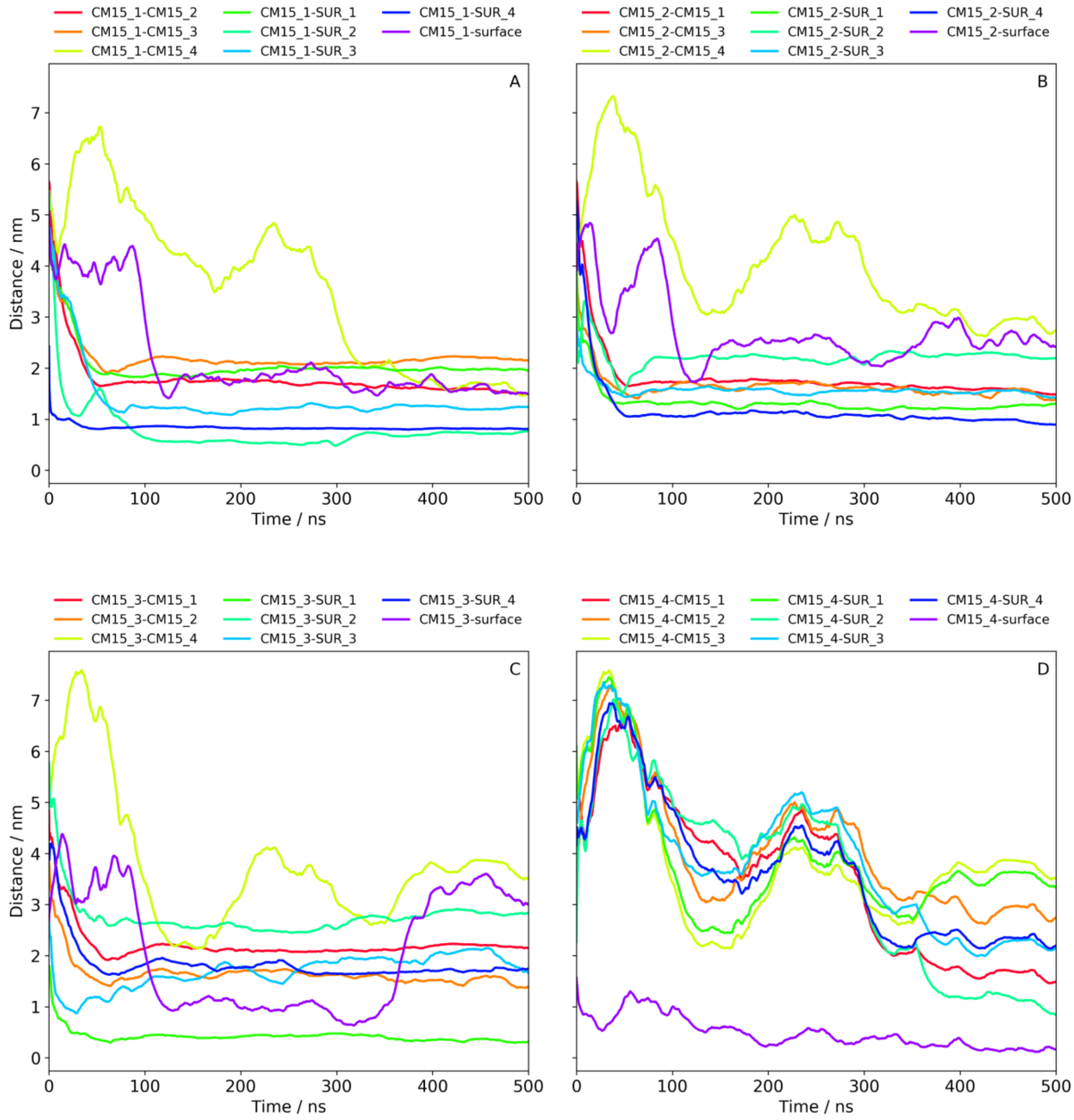

Figure S24. *CM15 distances* of the 4:4 CM15-suramin systems *in DOPC-DOPG environment*. The distances between the first (A), second (B), third (C) and fourth (D) CM15 molecule and all other component are shown.

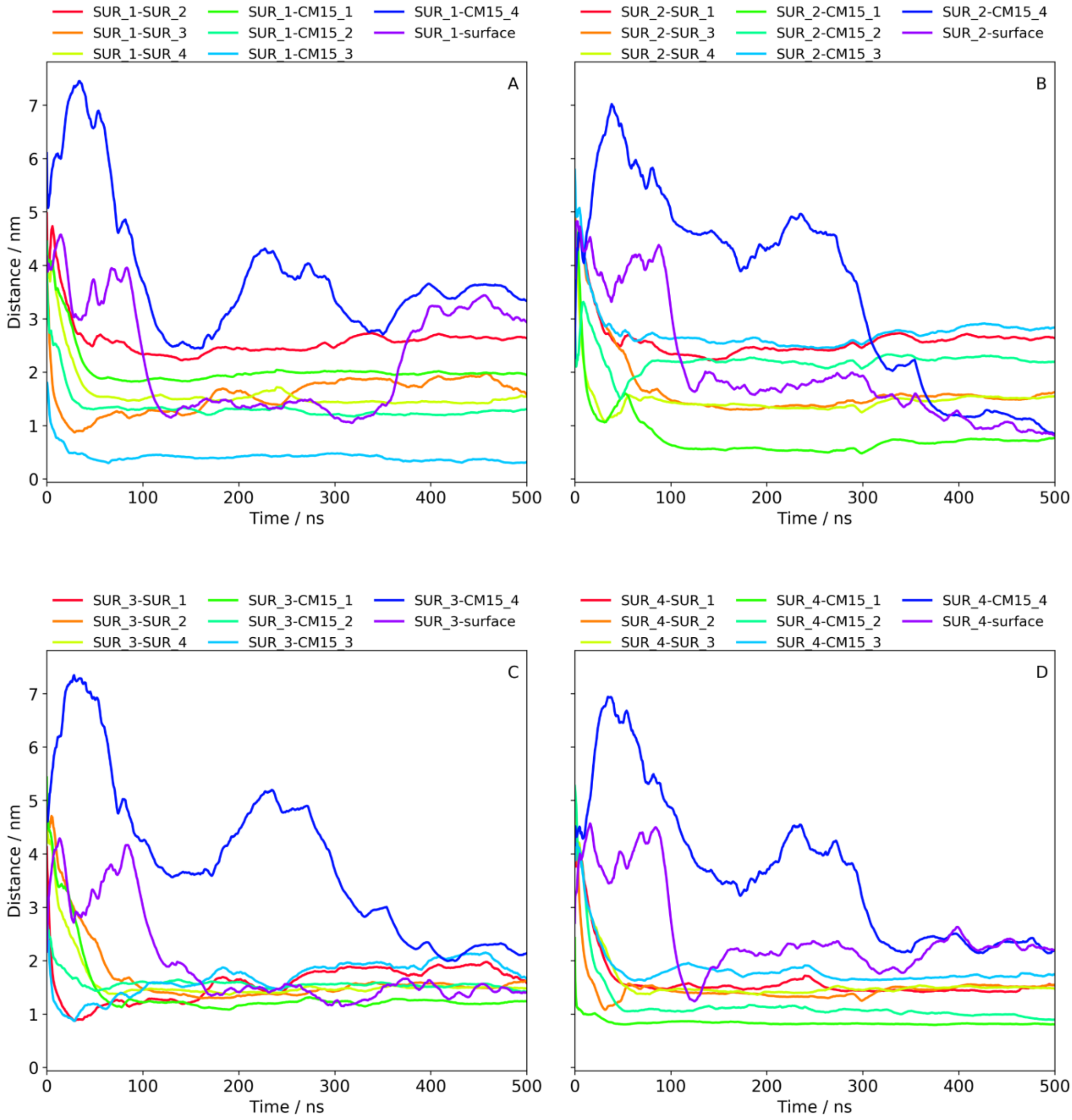

Figure S25. *Suramin distances* of the 4:4 CM15-suramin systems *in DOPC-DOPG environment*. The distances between the first (A), second (B), third (C) and fourth (D) suramin molecule and all other component are shown.

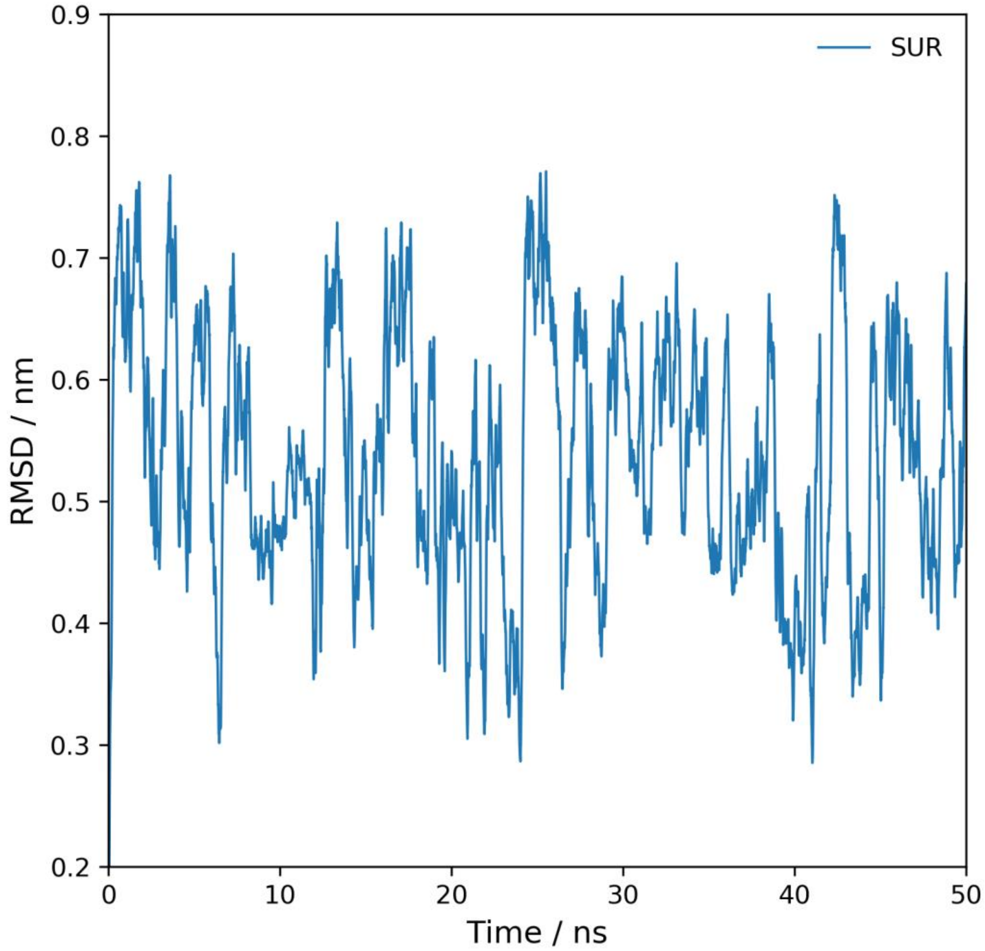

Figure S26. Root mean square deviation (RMSD) analysis of suramin in aqueous phase. 50 ns long all-atom simulation with one suramin molecule centered in a dodecahedron box with 1.5 nm from the box edges. The constantly changing curve indicates the high flexibility of the molecule in the aqueous phase.

Table S2. Excitation energies, wavelengths, and oscillator strengths of the calculated excited states.

State No.	Excitation Energy / eV	Wavelength / nm	Oscillator Strength
1	4.6884	264.45	0.0823
2	4.7064	263.44	0.0751
3	4.8240	257.01	0.2864
4	4.9112	252.45	0.3138
5	4.9484	250.56	0.0356
6	5.1050	242.87	0.0043
7	5.1472	240.88	0.1838
8	5.1641	240.09	0.0260
9	5.1807	239.32	0.7565
10	5.3512	231.69	0.2152

REFERENCES

(1) Paulo C T Souza; Marrink, S.-J. Martini 3 - Open Beta-Release, Http://Cgmartini.Nl. **2020**.

(2) Marrink, S. J.; Risselada, H. J.; Yefimov, S.; Tieleman, D. P.; de Vries, A. H. The MARTINI Force Field: Coarse Grained Model for Biomolecular Simulations. *J. Phys. Chem. B* **2007**, *111* (27), 7812–7824. https://doi.org/10.1021/jp071097f.

(3) Huang, J.; Rauscher, S.; Nawrocki, G.; Ran, T.; Feig, M.; Groot, B. L. de; Grubmüller, H.; Jr, A. D. M. CHARMM36m: An Improved Force Field for Folded and Intrinsically Disordered Proteins. *Nature Methods* **2017**, *14* (1), 71–73. https://doi.org/10.1038/nmeth.4067.

(4) Torrie, G. M.; Valleau, J. P. Nonphysical Sampling Distributions in Monte Carlo Free-Energy Estimation: Umbrella Sampling. *Journal of Computational Physics* **1977**, *23* (2), 187–199. https://doi.org/10.1016/0021-9991(77)90121-8.

(5) Tribello, G. A.; Bonomi, M.; Branduardi, D.; Camilloni, C.; Bussi, G. PLUMED 2: New Feathers for an Old Bird. *Computer Physics Communications* **2014**, *185* (2), 604–613. https://doi.org/10.1016/j.cpc.2013.09.018.

(6) Lee, T.-S.; Radak, B. K.; Pabis, A.; York, D. M. A New Maximum Likelihood Approach for Free Energy Profile Construction from Molecular Simulations. *J. Chem. Theory Comput.* **2013**, *9* (1), 153–164. https://doi.org/10.1021/ct300703z.
